# Osteoporosis and sarcopenia-related traits: A bi-directional Mendelian randomization study

**DOI:** 10.3389/fendo.2022.975647

**Published:** 2022-09-14

**Authors:** Chao Liu, Ningyuan Liu, Yu Xia, Ziyue Zhao, Tao Xiao, Hui Li

**Affiliations:** ^1^ Department of Orthopedics, the Second Xiangya Hospital of Central South University, Changsha, China; ^2^ Orthopedic Biomedical Materials Engineering Laboratory of Hunan Province, Changsha, China

**Keywords:** osteoporosis, sarcopenia, mendelian randomization, osteosarcopenia, aging

## Abstract

**Background:**

With the advancement of world population aging, age-related osteoporosis (OP) and sarcopenia (SP) impose enormous clinical and economic burden on society. Evidence from accumulating studies indicates that they mutually influence one another. However, an observational study may be affected by potential confounders. Meanwhile, a Mendelian randomization (MR) study can overcome these confounders to assess causality.

**Objectives:**

The aim of this study was to evaluate the causality between OP and SP, informing new strategies for prevention, diagnosis, and treatment of osteosarcopenia.

**Methods:**

Instrumental variables (IVs) at the genome‐wide significance level were obtained from published summary statistics, and the inverse variance weighted method and several other MR methods were conducted to evaluate the bi-directional causality between SP and OP. Myopia was analyzed as a negative control outcome to test the validity of IVs.

**Results:**

Femoral neck bone mineral density (FN BMD), lumbar spine BMD (LS BMD), and forearm BMD (FA BMD) had a direct causal effect on appendicular lean mass (ALM) [FA BMD-related analysis: odds ratio (OR) = 1.028, 95% confidence interval (CI) = (1.008,1.049), *p* = 0.006; FN BMD-related analysis: OR (95% CI) = 1.131 (1.092,1.170), *p* = 3.18E-12; LS BMD-related analysis: OR (95% CI) = 1.080 (1.062,1.098), *p* = 2.86E-19]. ALM had a significant causal effect on LS BMD [OR (95% CI) = (1.033,1.147), *p* = 0.001]. There was no evidence for causal association between BMD and low grip strength.

**Conclusions:**

OP and SP might mutually have a significant causal effect on each other. Our results supported the idea that the patient with severe OP was more susceptible to lose ALM and severe ALM loss might reduce LS BMD.

## Introduction

With the global aging of the population, the prevalence of osteoporosis (OP) and sarcopenia (SP) is increasing rapidly, which is positively associated with increased risk of fractures, reduced quality of life, and early death (1). They have caused a serious global public health problem, imposing enormous clinical and economic burden on society. OP and SP are both geriatric chronic disease with high incidence, and they are generally more prone to coexist in the same old person. Osteosarcopenia was proposed by Duque and colleagues to describe this overlap in 2017 (2). Among the community-dwelling older people, osteosarcopenia had widely ranging prevalence rates of approximately 5%–37% (≥65 years); meanwhile, osteosarcopenic individuals demonstrated poorer nutritional status than OP or SP alone (3). Considerable lines of evidence numerically indicated a close relationship between OP and SP on the basis of observational studies (4, 5). A recent meta-analysis study showed that OP was an associated factor of SP (6). However, two systematic reviews of RCTs reported that higher protein supplementation was only associated with lumbar spine bone mineral density (LS BMD) (7, 8). These conflicting results make it difficult to infer the causality between OP and SP, particularly when unmeasured potential confounders are involved, such as age, fat, and exercise, which can lead to OP and SP. Evaluating the causality between OP and SP can inform new strategies for prevention, diagnosis, and treatment of osteosarcopenia.

Mendelian randomization (MR) is a valid approach for causal inference using genetic variants as instrument variables (IVs), which can effectively overcome the confounding bias of traditional epidemiological studies (9). To the best of the authors’ knowledge, no MR has been investigated between OP and SP. Actually, nor did any randomized controlled trial (RCT) directly evaluate the bi-directional relation. Therefore, we performed a bi-directional two-sample MR analysis to address the associations between OP (measured as BMD) and SP (measured as body lean mass and low grip strength).

## Materials and methods

### Study design

**Figure 1:**
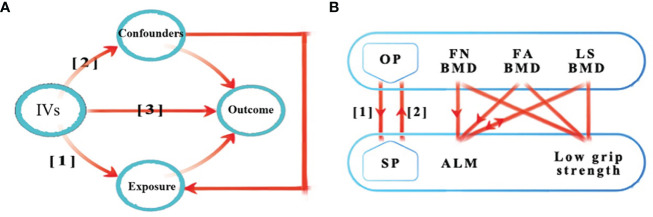
Schematic representation of the three assumptions and study design. **(A)** (1) The used genetic IVs are robustly associated with exposure; (2) The slected IVs are not associated with potential confounders; (3) The IVs can only affect the risk of outcome dependently through exposure. **(B)** This bidirectional MR analysis was performed in two steps: osteoporosis was studied as exposure while sarcopenia-related traits were studied as outcome in the first step, whereas the second step was reversed. The arrows indicate direction of causality in our results. IVs, instrumental variables; OP, osteoporosis; SP, sarcopenia; FA, forearm; FN, femoral neck; LS, lumbar spine; BMD, bone mineral density; ALM, appendicular lean mass; MR, mendelian randomization.

Valid MR analysis is based on three assumptions: (1) the used genetic IVs are robustly associated with exposure; (2) the selected IVs are not associated with potential confounders; and (3) the IVs can only affect the risk of outcome dependently through exposure (10). This bi-directional MR analysis was performed in two steps: OP was investigated as exposure while SP-related traits were investigated as outcome in the first step, whereas the second step was reversed. **Figure 1** shows an overview of the three assumptions and study design.

### Data sources

Clinically, femoral neck BMD (FN BMD), LS BMD, and forearm BMD (FA BMD) have been widely used as measurable and powerful predictors of OP. BMD is highly heritable and associated with common genetic variants (11). Related GWAS summary statistics were derived from the Genetic Factors for Osteoporosis (GEFOS) consortium (http://www.gefos.org/?q=content/data-release-2015) (12). The genetic values in 53,236 individuals of European ancestry were corrected for sex, age, age^2^, and weight, and standardized. Appendicular lean mass (ALM) is potentially important as a measure of muscle mass in older people (13). The ALM-related values were quantified by the sum of fat-free mass with 450,243 UK Biobank cohort participants (https://www.ebi.ac.uk/gwas/publications/33097823), adjusted for appendicular fat mass, age, age^2^, the top 10 principal components, and other covariates (14). The summary‐level statistics of low grip strength were obtained from a muscle weakness-related meta‐analysis study (https://www.ebi.ac.uk/gwas/publications/33510174) (15). The data included 256,523 individuals of European descent and were adjusted for sex, age, and population substructure. More details for phenotype and modeling, genotype quality control, and related association analysis can be found in the original publications (12, 14, 15).

### Genetic instrumental variable selection

In accordance with the three assumptions for MR analysis, independent single-nucleotide polymorphisms (SNPs) associated with the exposure at the genome‐wide significance level (*p* < 5 × 10^-8^) were selected as instrumental SNPs (clumping *r* ^2^ = 0.001 and kb = 10,000) (16). The association values of the corresponding SNPs were also obtained from outcome GWAS summary statistics. However, only three SNPs were selected for FA BMD so that we relaxed the criteria to 1 × 10^−6^ for selecting FA-related IVs. After harmonizing, we calculated the *F* statistic to evaluate the strength of selected IVs (https://sb452.shinyapps.io/overlap) (17). Genetic IV with *F* statistics >10 indicated a good strength of instrument to alleviate potential bias in MR analysis.

### MR analysis

The inverse variance weighted (IVW) method was performed to evaluate the bi-directional relation between SP and OP as the main statistical approach (https://mrcieu.github.io/TwoSampleMR/). The IVW method was considered as the most accurate method for estimating the causal relationship if no clear evidence for the presence of directional pleiotropy (*p* for MR-Egger intercept > 0.05) (18). When there was insufficient evidence of heterogeneity (*p* for MR-heterogeneity > 0.05) in these selected IVs, a random-effects model was conducted; otherwise, a fixed-effects model was assumed. A weighted median method was also conducted, which can generate effective causal estimates when at least 50% valid IVs were present in all the selected IVs (19). Robust Adjusted Profile Score (RAPS) could eliminate bias and assess causal relationship, even when there were hundreds of weak IVs (20). MR-PRESSO tested for pleiotropy and detected the outliers. After that, the IVW method was repeated (21). Considering multiple testing of OP and SP-related traits, we applied a conservative approach (Bonferroni) by adjusting the *p*-values (*p* = 0.05/(2×3) = 0.008).

### Sensitivity analysis

To test the robustness of our results, several sensitivity analyses were performed. Heterogeneity across IVs was evaluated by Cochran’s *Q* statistic. MR pleiotropy test was employed to perform MR Egger and returns intercept values to assess horizontal pleiotropy. The MR Steiger test was conducted to examine whether the assumption that exposure causes outcome was valid. Considering that various confounders were closely associated with the pathogenesis of OP and SP, we conservatively culled SNPs, which were closely related to whole-body fat mass, physical activity, and vitamin D levels at the genome‐wide significance level. Data related to confounders were derived from the GWAS Catalog (https://www.ebi.ac.uk/gwas) and GWAS summary data (https://gwas.mrcieu.ac.uk/). We repeated the MR analysis after excluding confounding SNPs.

### Negative control

To the best of the authors’ knowledge, there was no evidence of a link between myopia and OP or SP. Myopia was analyzed as a negative control outcome. Summary‐level data for myopia were obtained from the FinnGen biobank (https://www.finngen.fi/en), including 252,923 individuals of European ancestry.

All the MR tests were performed with the R packages “TwosampleMR”, “MendelianRandomization”, and “MRPRESSO” in the R statistical software (Version 4.1.2).

## Results

### Stage 1: Influence of osteoporosis on sarcopenia-related traits

In the first stage, the *F* statistics of FA BMD, FN BMD, and LS BMD were calculated and the results were 10.71, 33.38, and 26.41, respectively. Obviously, all the values were larger than 10, which indicated that the selected IVs were powerful enough to eliminate potential bias. The variance explained by the IVs we selected for FA BMD, FN BMD, and LS BMD was calculated through the MR Steiger test (**Supplementary Table 1**). The influence of OP on low grip strength was studied. A total of 15, 20, and 22 LD-independent and appropriate IVs were selected from GWASs for FA BMD, FN BMD, and LS BMD, respectively (**Supplementary Table 2**). As shown in **Table 1**, the IVW results suggested that BMD had no causal effect on low grip strength [FA BMD-related analysis: odds ratio (OR) = 1.006, 95% confidence interval (CI) = (0.945,1.071), *p* = 0.857; FN BMD-related analysis: OR (95% CI) = 0.971 (0.915,1.030), *p* = 0.322; LS BMD-related analysis: 0.988 (0.939,1.040), *p* = 0.655]. The MR pleiotropy test showed no horizontal pleiotropy and no outlier IV was identified in the MR‐PRESSO analysis. In total, all MR analyses supported the idea that OP had no significant causal effect on low grip strength.

**Table 1:** Mendelian randomization estimates for BMD on sarcopenia-related traits with all selected IVs.

Exposures	Outcomes	No. of IVs	Heterogeneity test	MR Egger	MR results		
			Cochran’s *Q* (*p*)	Intercept (*p*)	Method	OR (95% CI)	*p*
FA BMD	Low‐grip strength	15	28.881 (0.011)	0.004 (0.721)	IVW	1.006 (0.945,1.071)	0.857
					Weighted median	1.003 (0.938,1.073)	0.925
					RAPS	1.007 (0.945,1.073)	0.835
					MR-PRESSO (NA)	1.006 (0.945,1.071)	0.857
FN BMD	Low grip strength	20	21.530 (0.308)	0.009 (0.361)	IVW	0.971 (0.915,1.030)	0.322
					Weighted median	0.965 (0.888,1.049)	0.400
					RAPS	0.971 (0.911,1.036)	0.379
					MR-PRESSO (NA)	0.971 (0.915,1.030)	0.322
LS BMD	Low grip strength	22	27.780 (0.147)	0.003 (0.758)	IVW	0.988 (0.939,1.040)	0.655
					Weighted median	0.961 (0.893,1.035)	0.297
					RAPS	0.998 (0.941,1.058)	0.950
					MR-PRESSO (NA)	0.988 (0.939,1.040)	0.655
FA BMD	ALM	16	117.827 (<0.001)	5.95e-04 (0.901)	IVW	1.03 (0.999,1.062)	0.059
					Weighted median	1.026 (1.008,1.045)	**0.006**
					RAPS	1.026 (0.996,1.056)	0.085
					MR-PRESSO (2)	1.028 (1.008,1.049)	**0.006**
FN BMD	ALM	20	421.208 (<0.001)	0.028 (0.010)	IVW	1.124 (1.047,1.208)	**0.001**
					Weighted median	1.093 (1.056,1.131)	**5.08E-07**
					RAPS	1.100 (1.018,1,188)	0.016
					MR-PRESSO (8)	1.131 (1.092,1.170)	**3.18E-12**
LS BMD	ALM	22	245.869 (<0.001)	0.005 (0.466)	IVW	1.090 (1.043,1.140)	**1.53E-04**
					Weighted median	1.069 (1.044,1.095)	**2.10E-08**
					RAPS	1.075 (1.049,1.101)	**3.20E-09**
					MR-PRESSO (8)	1.080 (1.062,1.098)	**2.86E-19**

Numbers in parentheses depict outlier IVs number (**Supplementary Table 3**).

Bonferroni-corrected significance level (0.05/(2×3) = 0.008) was used to correct for multiple comparisons. p < 0.008. The bold values meant that the p < 0.008.

BMD, bone mineral density; ALM, appendicular lean mass; FA, forearm; FN, femoral neck; LS, lumbar spine; MR, mendelian randomization; IVs, instrumental variables; CI, confidence interval; OR, odds ratio; IVW, inverse variance weighted; MR-PRESSO, Mendelian Randomization Pleiotropy RESidual Sum and Outlier; MR-RAPS, Mendelian Randomization Robust Adjusted Profile Score.

The influence of OP on ALM was also studied. A total of 16, 20, and 22 IVs were obtained for FA BMD, FN BMD, and LS BMD, respectively (**Supplementary Table 2**). The MR pleiotropy test detected horizontal pleiotropy in FN BMD-related IVs (intercept = 0.028, *p* = 0.010) and MR-PRESSO detected several potential pleiotropic IVs for BMD (**Supplementary Table 3**). After the outliers were removed, the IVW results indicated that BMD had a significant causal effect on ALM [FA BMD-related analysis: OR (95% CI) = 1.028 (1.008,1.049), *p* = 0.006; FA BMD-related analysis: OR (95% CI) = 1.131 (1.092,1.170), *p* = 3.18E-12; LS BMD-related analysis: OR (95% CI) = 1.080 (1.062,1.098), *p* = 2.86E-19]. Other MR analysis results are shown in **Table 1**. In total, most MR analyses supported the notion that OP had a significant negative causal effect on ALM.

In stage 1, no IV was removed because they had no intersection with confounding SNPs (**Supplementary Table 4**).

### Stage 2: Influence of sarcopenia-related traits on osteoporosis

In the second stage, the *F* statistics of low grip strength and ALM were computed and the results were 43.70 and 17.22, respectively. The variance explained by the IVs we selected for low grip strength and ALM were provided in **Supplementary Table 1**.

The influence of low grip strength on OP was studied. A total of 10, 10, and 10 LD-independent IVs at the genome‐wide significance level were selected from GWASs for low grip strength (**Supplementary Table 5**). As shown in **Table 2**, the IVW results suggested that low grip strength had no causal effect on OP [FA BMD-related analysis: OR (95% CI) = 1.191 (0.943,1.505), *p* = 0.142; FN BMD-related analysis: OR (95% CI) = 0.952 (0.811,1.116), *p* = 0.543; LS BMD-related analysis: 0.887 (0.776,1.013), *p* = 0.077]. The MR pleiotropy test showed no horizontal pleiotropy and no outlier IV was identified in the MR‐PRESSO analysis. In total, all MR analyses supported the idea that low grip strength had no significant causal effect on OP.

**Table 2:** Mendelian randomization estimates for sarcopenia-related traits on BMD with all selected IVs.

Exposures	Outcomes	No. of IVs	Heterogeneity test	MR Egger	MR results		
			Cochran’s *Q* (*p*)	Intercept (*p*)	Method	OR (95% CI)	*p*
Low grip strength	FA BMD	10	7.743 (0.560)	0.015 (0.458)	IVW	1.191 (0.943,1.505)	0.142
					Weighted median	1.124 (0.815,1.549)	0.477
					RAPS	1.238 (0.977,1.568)	0.077
					MR-PRESSO (NA)	1.191 (0.943,1.505)	0.142
Low grip strength	FN BMD	10	17.196 (0.046)	0.009 (0.531)	IVW	0.952 (0.811,1.116)	0.543
					Weighted median	0.935 (0.791,1.106)	0.436
					RAPS	0.888 (0.720,1.095)	0.267
					MR-PRESSO (NA)	0.952 (0.811,1.116)	0.543
Low grip strength	LS BMD	10	15.696 (0.074)	0.002 (0.905)	IVW	0.887 (0.776,1.013)	0.077
					Weighted median	0.853 (0.710,1.024)	0.089
					RAPS	0.856 (0.748,0.980)	0.024
					MR-PRESSO (NA)	0.887 (0.776,1.013)	0.077
ALM	FA BMD	562	631.596 (0.020)	0.002 (0.420)	IVW	0.957 (0.888,1.031)	0.245
					Weighted median	0.931 (0.834,1.040)	0.208
					RAPS	0.931 (0.864,1.003)	0.058
					MR-PRESSO (1)	0.951 (0.886,1.021)	0.163
ALM	FN BMD	520	877.962 (<0.001)	0.002 (0.199)	IVW	1.011 (0.965,1.059)	0.650
					Weighted median	0.981 (0.923,1.043)	0.546
					RAPS	1.003 (0.960,1.047)	0.909
					MR-PRESSO (5)	0.988 (0.947,1.031)	0.589
ALM	LS BMD	519	808.448 (<0.001)	6.41e-04 (0.646)	IVW	1.088 (1.033,1.147)	**0.001**
					Weighted median	1.059 (0.990,1.133)	0.093
					RAPS	1.071 (1.020,1.124)	**0.006**
					MR-PRESSO (3)	1.068 (1.018,1.121)	**0.007**

Numbers in parentheses depict outlier IVs number (**Supplementary Table 3**).

Bonferroni-corrected significance level (0.05/(2×3) = 0.008) was used to correct for multiple comparisons. p < 0.008. The bold values meant that the p < 0.008.

BMD, bone mineral density; ALM, appendicular lean mass; FA, forearm; FN, femoral neck; LS, lumbar spine; MR, mendelian randomization; IVs, instrumental variables; CI, confidence interval; OR, odds ratio; IVW, inverse variance weighted; MR-PRESSO, Mendelian Randomization Pleiotropy RESidual Sum and Outlier; MR-RAPS, Mendelian Randomization Robust Adjusted Profile Score.

The influence of ALM on OP was also studied. A total of 560, 520, and 519 appropriate IVs were obtained for ALM, respectively (**Supplementary Table 5**). The MR pleiotropy test showed no horizontal pleiotropy, but MR-PRESSO detected several potential pleiotropic IVs for ALM (**Supplementary Table 3**). In total, combined with the MR results detailed above, the results of the MR analyses supported the notion that ALM had no significant causal effect on FA BMD or FN BMD while it identified a significant negative causal effect of ALM on LS BMD, consistent with the IVW results [FA BMD-related analysis: OR (95% CI) = 0.957 (0.888,1.031), *p* = 0.245; FN BMD-related analysis: OR (95% CI) = 1.011 (0.965,1.059), *p* = 0.650; LS BMD-related analysis: 1.088 (1.033,1.147), *p* = 0.001].

In stage 2, several IVs were removed because they had a significant intersection with confounding SNPs (**Supplementary Table 4**). However, the significance of MR analysis results and horizontal pleiotropy were exactly the same as before (**Table 3**). The negative control analysis results indicated that FA BMD, FN BMD, LS BMD, low grip strength, and ALM were not relevant to myopia such that the IVs we selected were appropriate (**Supplementary Tables 1**, **6**, and **7**).

**Table 3:** Mendelian randomization estimates for sarcopenia-related traits on BMD after removing confounding IVs.

Exposures	Outcomes	No. of IVs	Heterogeneity test	MR Egger	MR results		
			Cochran’s *Q* (*p*)	Intercept (*p*)	Method	OR (95% CI)	*p*
Low grip strength	FA BMD	9	6.791 (0.559)	0.010 (0.645)	IVW	1.145 (0.895,1.466)	0.281
					Weighted median	1.120 (0.804,1.560)	0.502
					RAPS	1.190 (0.929,1.525)	0.168
					MR-PRESSO (NA)	1.145 (0.895,1.466)	0.281
Low grip strength	FN BMD	9	16.230 (0.039)	0.0068 (0.679)	IVW	0.933 (0.784,1.110)	0.433
					Weighted median	0.910 (0.761,1.089)	0.304
					RAPS	0.870 (0.696,1.087)	0.220
					MR-PRESSO (NA)	0.933 (0.784,1.110)	0.433
Low grip strength	LS BMD	9	15.653 (0.048)	0.003 (0.870)	IVW	0.891 (0.731,1.085)	0.251
					Weighted median	0.882 (0.730,1.066)	0.193
					RAPS	0.867 (0.737,1.020)	0.085
					MR-PRESSO (NA)	0.891 (0.731,1.085)	0.251
ALM	FA BMD	523	588.251 (0.023)	−0.002 (0.425)	IVW	0.973 (0.899,1.054)	0.502
					Weighted median	0.973 (0.864,1.096)	0.652
					RAPS	0.945 (0.874,1.022)	0.156
					MR-PRESSO (1)	0.967 (0.897,1.042)	0.377
ALM	FN BMD	486	810.902 (<0.001)	5.26e-04 (0.693)	IVW	1.029 (0.979,1.080)	0.258
					Weighted median	1.010 (0.949,1.075)	0.76
					RAPS	1.016 (0.971,1.062)	0.491
					MR-PRESSO (6)	1.007 (0.969,1.046)	0.718
ALM	LS BMD	485	749.745 (<0.001)	−5.96e-04 (0.689)	IVW	1.109 (1.050,1.171)	**2.15e-04**
					Weighted median	1.063 (0.989,1.143)	0.095
					RAPS	1.082 (1.028,1.139)	**0.003**
					MR-PRESSO (3)	1.086 (1.033,1.142)	**0.001**

Numbers in parentheses depict outlier IVs number (**Supplementary Table 3**).

Bonferroni-corrected significance level (0.05/(2×3) = 0.008) was used to correct for multiple comparisons. p < 0.008. The bold values meant that the p < 0.008.

BMD, bone mineral density; ALM, appendicular lean mass; FA, forearm; FN, femoral neck; LS, lumbar spine; MR, mendelian randomization; IVs, instrumental variables; CI, confidence interval; OR, odds ratio; IVW, inverse variance weighted; MR-PRESSO, Mendelian Randomization Pleiotropy RESidual Sum and Outlier; MR-RAPS, Mendelian Randomization Robust Adjusted Profile Score.

## Discussion

Based on our results, we successfully concluded that OP and SP might mutually had a significant causal effect on each other, identifying the significant positive causal effect of FA BMD, FN BMD, and LS BMD on ALM and the significant positive causal effect of ALM on LS BMD. However, there was no evidence for causal association between BMD and low grip strength. To our knowledge, this is the first bi-directional MR study to investigate causality between OP and SP, considering potential confounders.

Despite these differences, two studies with similar themes at the genetic level are present. It was observed that there was no significant genetic correlation between low grip strength and osteoporotic fracture risk after multiple-testing correction (15), whose result was not in conflict with our assessment of the causality between OP and low grip strength. It was worth noting that low grip strength was a major criterion in SP definition, which indicated that the causality between OP and SP was only partially proved in our results. Additional studies with better MR methods and data would be needed to verify the causality between OP and low grip strength in the future. Pei briefly conducted an MR analysis predicting a causal effect of ALM on fracture (14) while our result indicated that ALM had a significant causal effect on LS BMD, but not FA or FN BMD. Considering that protein supplementation can effectively increase lean mass, we broadened our search to explore more RCT evidence (22). Two meta-analyses involving RCTs and prospective cohort studies suggested that higher compared with lower protein intake had a protective effect on LS BMD, but no effect on total hip, femoral neck, or total body BMD (7). Our conclusion so far is consistent with these findings of relevant studies at the gene level or RCT.

Many previous observation studies have demonstrated the positive correlation between SP and OP (23–25). According to data from the OsteoSys study, 90% of the sarcopenic patients demonstrated low BMD while only few patients with low BMD demonstrated SP (24). A prospective study of 168,682 UK biobank participants demonstrated that pre-sarcopenic men and sarcopenic women had a higher risk of developing OP (25). Our bi-directional MR study further complements previous studies and provided evidence of causality between OP and SP. Bone and muscle are closely connected spatially, and mechanical signals are transmitted from muscle strength to coordinate BMD and muscle mass (26). A recent retrospective study also indicated that chair rising test maximum force and grip strength were positively correlated with cortical geometric and microarchitectural parameters at all measured sites (27). Moreover, accumulating evidence suggested that bone and muscle can secrete a variety of cytokines to modulate each other, including myostatin, irisin, interleukin 6, osteocalcin, RANKL, and osteoprotegerin (28). Notably, as muscle ages, pathophysiological processes in muscle function present as selective loss of fast motor neurons while progressive loss of skeletal muscle mass presents as atrophy of muscle fibers, loss of number of muscle fibers, and reduced number of satellite cells (29). However, a large study including 20,400 adults aged 60 years and over showed that telomere length was not associated with low ALM, low BMD, or low grip strength (30). Anyways, resistance and endurance exercises, creatine monohydrate supplementation, and intake of protein and vitamin D have protective effects on aging muscle and bone (31, 32). Our MR result indicated that OP had a significant causal effect on ALM instead of low grip strength, which provided more information on the mechanisms of muscle–bone crosstalk.

This study is the first bi-directional MR study to investigate causality between OP and SP. Several MR analysis methods were performed to ensure the accuracy and validity of our results; finally, largely consistent results were obtained, which made our results more reliable. The additional negative control, combined with the MR Steiger test, was incorporated to ensure the validity of IVs we selected. Furthermore, to satisfy the second assumptions of MR, we conservatively culled confounding SNPs and the conclusions remain the same and valid. Nevertheless, this study still has several potential limitations. Only the summary‐level statistics were extracted so that we did not evaluate the effect depending on different age and gender separately. Our results indicated that individuals with OP were prone to lose ALM and that severe ALM loss could reduce LS BMD. Meanwhile, it also showed that ALM had no significant causal effect on FA BMD or FN BMD, which warranted scrupulous consideration. Further MR studies with a larger sample size or RCTs are needed to obtain more accurate results. Although three main confounders were removed, other confounders may still work through the second assumptions of MR. Malnutrition should also be considered, which could help us understand the causality between OP and SP in our study. Regrettably, we did not find a qualified statistic of malnutrition. Considering that we should not infer causality from correlation, we only conservatively culled confounding SNPs at the genome‐wide significance level in the absence of relevant data support.

## Conclusions

In conclusion, OP and SP might mutually have a significant causal effect on each other. We identified the significant positive causal effect of FA BMD, FN BMD, and LS BMD on ALM and the significant positive causal effect of ALM on LS BMD. There was no evidence for the causal association between BMD and low grip strength.

## Data availability statement

The datasets presented in this study can be found in online repositories. The websites for these datasets have been provided in the article.

## Author contributions

CL had the idea and drafted the final manuscript. NYL performed data analysis. YX created the figure. YZZ gave constructive suggestions during the process. TX and HL drafted the final manuscript and finally approved the version to be published. All authors agreed to be accountable for all aspects of the work.

## Funding

This work was supported by Research Project of Human Health Commission (grant number 202204073071).

## Acknowledgments

All data used in this study were obtained from openly available databases and consortiums. We express our sincere appreciation to them.

## Conflict of interest

The authors declare that the research was conducted in the absence of any commercial or financial relationships that could be construed as a potential conflict of interest.

## Publisher’s note

All claims expressed in this article are solely those of the authors and do not necessarily represent those of their affiliated organizations, or those of the publisher, the editors and the reviewers. Any product that may be evaluated in this article, or claim that may be made by its manufacturer, is not guaranteed or endorsed by the publisher.
