# Supplementary material for: Osteoporosis and sarcopenia-related traits: A bi-directional Mendelian randomization study
Source: Front Endocrinol (Lausanne). 2022 Sep 14;13:975647. doi: 10.3389/fendo.2022.975647 (PMC9515352; doi:10.3389/fendo.2022.975647)
Supplement: Supplementary file 1 [file Table_1.docx]

Supplementary Table 1. Variance explained by IVs in MR-Steiger analysis.

| Exposure | outcome | snp_r2. exposure | snp_r2. outcome | MR-Steiger |
| --- | --- | --- | --- | --- |
| FA BMD | low‐grip strength | 0.057 | 1.13E-04 | TRUE |
| FA BMD | ALM | 0.057 | 3.24E-04 | TRUE |
| FN BMD | low‐grip strength | 0.031 | 8.79E-05 | TRUE |
| FN BMD | ALM | 0.031 | 1.00E-03 | TRUE |
| LS BMD | low‐grip strength | 0.038 | 1.14E-04 | TRUE |
| LS BMD | ALM | 0.038 | 1.00E-03 | TRUE |
| low‐grip strength | FA BMD | 0.002 | 1.00E-03 | TRUE |
| low‐grip strength | FN BMD | 0.002 | 1.00E-03 | TRUE |
| low‐grip strength | LS BMD | 0.002 | 7.13E-04 | TRUE |
| ALM | FA BMD | 0.126 | 8.20E-02 | TRUE |
| ALM | FN BMD | 0.115 | 2.80E-02 | TRUE |
| ALM | LS BMD | 0.115 | 3.10E-02 | TRUE |
| low‐grip strength | myopia | 0.002 | 9.30E-05 | TRUE |
| ALM | myopia | 0.129 | 3.00E-03 | TRUE |
| FA BMD | myopia | 0.001 | 7.23E-05 | TRUE |
| FN BMD | myopia | 0.029 | 7.56E-05 | TRUE |
| LS BMD | myopia | 0.037 | 1.11E-04 | TRUE |

BMD: bone mineral density; ALM: appendicular lean mass; FA: forearm; FN: femoral neck; LS: lumbar spine; IVs: instrumental variables.
