# Supplementary material for: Osteoporosis and sarcopenia-related traits: A bi-directional Mendelian randomization study
Source: Front Endocrinol (Lausanne). 2022 Sep 14;13:975647. doi: 10.3389/fendo.2022.975647 (PMC9515352; doi:10.3389/fendo.2022.975647)
Supplement: Supplementary file 2 [file Table_2.docx]

Supplementary Table 2. Independent IVs of FA BMD (g/cm^3^), FN BMD (g/cm^3^) and LS BMD (g/cm^3^) in stage 1 analysis.

| SNP | exposure | outcome | beta.exposure | beta.outcome | se.exposure | se.outcome | pval.exposure | pval.outcome | mr_keep |
| --- | --- | --- | --- | --- | --- | --- | --- | --- | --- |
| rs10801580 | FA BMD | low‐grip strength | 0.089 | 0.007 | 0.019 | 0.009 | 2.58E-06 | 0.422 | TRUE |
| rs1336819 | FA BMD | low‐grip strength | -0.234 | -0.017 | 0.049 | 0.024 | 3.17E-06 | 0.471 | TRUE |
| rs13423976 | FA BMD | low‐grip strength | -0.098 | -0.004 | 0.017 | 0.008 | 2.30E-08 | 0.650 | TRUE |
| rs145750983 | FA BMD | low‐grip strength | -0.299 | 0.010 | 0.063 | 0.028 | 2.87E-06 | 0.725 | TRUE |
| rs1982763 | FA BMD | low‐grip strength | 0.079 | 0.004 | 0.016 | 0.007 | 1.11E-06 | 0.622 | TRUE |
| rs35936330 | FA BMD | low‐grip strength | 0.095 | -0.015 | 0.020 | 0.010 | 3.30E-06 | 0.120 | TRUE |
| rs489247 | FA BMD | low‐grip strength | -0.086 | 0.009 | 0.018 | 0.008 | 2.90E-06 | 0.311 | TRUE |
| rs58715565 | FA BMD | low‐grip strength | -0.174 | 0.031 | 0.035 | 0.020 | 1.24E-06 | 0.127 | TRUE |
| rs61066067 | FA BMD | low‐grip strength | -0.138 | -0.020 | 0.027 | 0.013 | 5.87E-07 | 0.111 | TRUE |
| rs61904896 | FA BMD | low‐grip strength | 0.154 | 0.014 | 0.031 | 0.014 | 8.45E-07 | 0.324 | TRUE |
| rs6894139 | FA BMD | low‐grip strength | -0.089 | 0.018 | 0.016 | 0.007 | 2.52E-08 | 0.015 | TRUE |
| rs7216991 | FA BMD | low‐grip strength | -0.074 | -0.014 | 0.016 | 0.007 | 4.87E-06 | 0.066 | TRUE |
| rs7555157 | FA BMD | low‐grip strength | 0.084 | 0.024 | 0.018 | 0.009 | 3.75E-06 | 0.005 | TRUE |
| rs7776725 | FA BMD | low‐grip strength | 0.186 | -0.003 | 0.017 | 0.008 | 1.21E-25 | 0.699 | TRUE |
| rs7815608 | FA BMD | low‐grip strength | -0.081 | 0.006 | 0.017 | 0.008 | 4.34E-06 | 0.451 | TRUE |
| rs10170839 | FN BMD | low‐grip strength | -0.059 | 0.002 | 0.008 | 0.007 | 1.20E-14 | 0.746 | TRUE |
| rs10794639 | FN BMD | low‐grip strength | -0.051 | 0.010 | 0.008 | 0.007 | 3.30E-11 | 0.194 | TRUE |
| rs10946458 | FN BMD | low‐grip strength | -0.045 | -0.002 | 0.008 | 0.008 | 3.63E-08 | 0.773 | TRUE |
| rs11652763 | FN BMD | low‐grip strength | 0.084 | -0.001 | 0.013 | 0.013 | 1.09E-10 | 0.963 | TRUE |
| rs13194508 | FN BMD | low‐grip strength | -0.052 | 0.007 | 0.009 | 0.009 | 1.30E-08 | 0.412 | TRUE |
| rs1366594 | FN BMD | low‐grip strength | -0.079 | 0.017 | 0.008 | 0.007 | 5.44E-25 | 0.021 | TRUE |
| rs1485307 | FN BMD | low‐grip strength | 0.062 | 0.008 | 0.008 | 0.007 | 2.49E-15 | 0.268 | TRUE |
| rs1785493 | FN BMD | low‐grip strength | -0.045 | -0.004 | 0.008 | 0.008 | 4.06E-08 | 0.594 | TRUE |
| rs2566752 | FN BMD | low‐grip strength | 0.062 | -0.004 | 0.008 | 0.008 | 3.65E-15 | 0.638 | TRUE |
| rs2741856 | FN BMD | low‐grip strength | 0.088 | -0.022 | 0.014 | 0.014 | 1.34E-09 | 0.107 | TRUE |
| rs3779381 | FN BMD | low‐grip strength | 0.058 | -0.004 | 0.009 | 0.008 | 2.87E-11 | 0.613 | TRUE |
| rs4281029 | FN BMD | low‐grip strength | 0.057 | 0.004 | 0.009 | 0.009 | 2.96E-09 | 0.656 | TRUE |
| rs436448 | FN BMD | low‐grip strength | -0.064 | -0.006 | 0.008 | 0.007 | 1.56E-16 | 0.402 | TRUE |
| rs4448201 | FN BMD | low‐grip strength | -0.066 | 0.004 | 0.008 | 0.008 | 4.37E-16 | 0.621 | TRUE |
| rs4759320 | FN BMD | low‐grip strength | -0.045 | 0.015 | 0.008 | 0.008 | 3.33E-08 | 0.054 | TRUE |
| rs7108738 | FN BMD | low‐grip strength | 0.083 | -0.002 | 0.010 | 0.010 | 8.07E-17 | 0.836 | TRUE |
| rs71390846 | FN BMD | low‐grip strength | -0.059 | 0.003 | 0.010 | 0.010 | 3.16E-09 | 0.755 | TRUE |
| rs7209460 | FN BMD | low‐grip strength | -0.051 | -0.007 | 0.008 | 0.008 | 1.35E-09 | 0.360 | TRUE |
| rs7524102 | FN BMD | low‐grip strength | 0.084 | 0.002 | 0.010 | 0.010 | 7.36E-17 | 0.866 | TRUE |
| rs9478217 | FN BMD | low‐grip strength | -0.053 | -0.015 | 0.008 | 0.008 | 1.23E-11 | 0.041 | TRUE |
| rs1023940 | LS BMD | low‐grip strength | 0.065 | 0.002 | 0.009 | 0.007 | 6.47E-13 | 0.810 | TRUE |
| rs11002249 | LS BMD | low‐grip strength | 0.070 | 0.011 | 0.011 | 0.010 | 1.01E-09 | 0.256 | TRUE |
| rs11024028 | LS BMD | low‐grip strength | 0.066 | 0.009 | 0.011 | 0.009 | 1.44E-08 | 0.335 | TRUE |
| rs11680288 | LS BMD | low‐grip strength | 0.054 | -0.004 | 0.009 | 0.007 | 3.12E-09 | 0.562 | TRUE |
| rs11692564 | LS BMD | low‐grip strength | 0.238 | -0.030 | 0.039 | 0.029 | 4.10E-09 | 0.313 | TRUE |
| rs13046645 | LS BMD | low‐grip strength | -0.056 | -0.019 | 0.010 | 0.008 | 2.92E-08 | 0.022 | TRUE |
| rs1357651 | LS BMD | low‐grip strength | -0.068 | 0.017 | 0.009 | 0.008 | 3.75E-13 | 0.031 | TRUE |
| rs2220189 | LS BMD | low‐grip strength | 0.083 | 0.006 | 0.009 | 0.007 | 4.25E-20 | 0.453 | FALSE |
| rs2235811 | LS BMD | low‐grip strength | -0.054 | 0.005 | 0.009 | 0.007 | 4.66E-09 | 0.467 | TRUE |
| rs2291467 | LS BMD | low‐grip strength | -0.077 | -0.007 | 0.010 | 0.008 | 9.64E-14 | 0.439 | TRUE |
| rs2566752 | LS BMD | low‐grip strength | 0.083 | -0.004 | 0.009 | 0.008 | 1.49E-19 | 0.638 | TRUE |
| rs35681117 | LS BMD | low‐grip strength | 0.055 | 0.010 | 0.010 | 0.008 | 2.39E-08 | 0.221 | TRUE |
| rs401680 | LS BMD | low‐grip strength | -0.057 | -0.007 | 0.009 | 0.007 | 3.70E-10 | 0.349 | FALSE |
| rs6965122 | LS BMD | low‐grip strength | -0.062 | 0.006 | 0.009 | 0.008 | 7.40E-11 | 0.426 | TRUE |
| rs71390846 | LS BMD | low‐grip strength | -0.064 | 0.003 | 0.011 | 0.010 | 3.80E-08 | 0.755 | TRUE |
| rs73326583 | LS BMD | low‐grip strength | 0.072 | -0.003 | 0.012 | 0.010 | 2.83E-09 | 0.812 | TRUE |
| rs7524102 | LS BMD | low‐grip strength | 0.090 | 0.002 | 0.011 | 0.010 | 2.41E-14 | 0.866 | TRUE |
| rs7807953 | LS BMD | low‐grip strength | 0.075 | -0.006 | 0.010 | 0.008 | 4.11E-14 | 0.465 | TRUE |
| rs78667121 | LS BMD | low‐grip strength | 0.150 | 0.026 | 0.026 | 0.021 | 1.21E-08 | 0.223 | TRUE |
| rs884205 | LS BMD | low‐grip strength | -0.062 | -0.016 | 0.010 | 0.009 | 2.77E-09 | 0.059 | TRUE |
| rs894738 | LS BMD | low‐grip strength | -0.063 | 0.013 | 0.009 | 0.008 | 2.00E-11 | 0.099 | TRUE |
| rs9533094 | LS BMD | low‐grip strength | -0.083 | 0.000 | 0.009 | 0.007 | 2.80E-20 | 0.979 | TRUE |
| rs9749364 | LS BMD | low‐grip strength | 0.114 | -0.012 | 0.018 | 0.013 | 6.64E-10 | 0.375 | TRUE |
| rs9921222 | LS BMD | low‐grip strength | -0.053 | 0.010 | 0.009 | 0.007 | 3.16E-09 | 0.193 | TRUE |
| rs10801580 | FA BMD | ALM | 0.089 | 0.007 | 0.019 | 0.002 | 2.58E-06 | 0.001 | TRUE |
| rs115419274 | FA BMD | ALM | 0.451 | 0.028 | 0.097 | 0.012 | 4.90E-06 | 0.025 | TRUE |
| rs1336819 | FA BMD | ALM | -0.234 | -0.002 | 0.049 | 0.006 | 3.17E-06 | 0.703 | TRUE |
| rs13423976 | FA BMD | ALM | -0.098 | -0.002 | 0.017 | 0.002 | 2.30E-08 | 0.393 | TRUE |
| rs145750983 | FA BMD | ALM | -0.299 | 0.007 | 0.063 | 0.007 | 2.87E-06 | 0.288 | TRUE |
| rs1982763 | FA BMD | ALM | 0.079 | 0.007 | 0.016 | 0.002 | 1.11E-06 | 0.000 | TRUE |
| rs35936330 | FA BMD | ALM | 0.095 | 0.009 | 0.020 | 0.002 | 3.30E-06 | 0.000 | TRUE |
| rs489247 | FA BMD | ALM | -0.086 | -0.003 | 0.018 | 0.002 | 2.90E-06 | 0.145 | TRUE |
| rs58715565 | FA BMD | ALM | -0.174 | -0.006 | 0.035 | 0.005 | 1.24E-06 | 0.238 | TRUE |
| rs61066067 | FA BMD | ALM | -0.138 | 0.001 | 0.027 | 0.003 | 5.87E-07 | 0.690 | TRUE |
| rs61904896 | FA BMD | ALM | 0.154 | 0.003 | 0.031 | 0.004 | 8.45E-07 | 0.394 | TRUE |
| rs6894139 | FA BMD | ALM | -0.089 | -0.014 | 0.016 | 0.002 | 2.52E-08 | 0.000 | TRUE |
| rs7216991 | FA BMD | ALM | -0.074 | 0.010 | 0.016 | 0.002 | 4.87E-06 | 0.000 | TRUE |
| rs7555157 | FA BMD | ALM | 0.084 | -0.004 | 0.018 | 0.002 | 3.75E-06 | 0.101 | TRUE |
| rs7776725 | FA BMD | ALM | 0.186 | 0.006 | 0.017 | 0.002 | 1.21E-25 | 0.007 | TRUE |
| rs7815608 | FA BMD | ALM | -0.081 | 0.001 | 0.017 | 0.002 | 4.34E-06 | 0.584 | TRUE |
| rs10170839 | FN BMD | ALM | -0.059 | -0.004 | 0.008 | 0.002 | 1.20E-14 | 0.022 | TRUE |
| rs10794639 | FN BMD | ALM | -0.051 | -0.012 | 0.008 | 0.002 | 3.30E-11 | 0.000 | TRUE |
| rs10946458 | FN BMD | ALM | -0.045 | -0.002 | 0.008 | 0.002 | 3.63E-08 | 0.375 | TRUE |
| rs11652763 | FN BMD | ALM | 0.084 | -0.009 | 0.013 | 0.003 | 1.09E-10 | 0.004 | TRUE |
| rs13194508 | FN BMD | ALM | -0.052 | -0.032 | 0.009 | 0.002 | 1.30E-08 | 0.000 | TRUE |
| rs1366594 | FN BMD | ALM | -0.079 | -0.015 | 0.008 | 0.002 | 5.44E-25 | 0.000 | TRUE |
| rs1485307 | FN BMD | ALM | 0.062 | 0.008 | 0.008 | 0.002 | 2.49E-15 | 0.000 | TRUE |
| rs1785493 | FN BMD | ALM | -0.045 | -0.019 | 0.008 | 0.002 | 4.06E-08 | 0.000 | TRUE |
| rs2566752 | FN BMD | ALM | 0.062 | 0.002 | 0.008 | 0.002 | 3.65E-15 | 0.447 | TRUE |
| rs2741856 | FN BMD | ALM | 0.088 | 0.008 | 0.014 | 0.004 | 1.34E-09 | 0.027 | TRUE |
| rs3779381 | FN BMD | ALM | 0.058 | 0.008 | 0.009 | 0.002 | 2.87E-11 | 0.000 | TRUE |
| rs4281029 | FN BMD | ALM | 0.057 | 0.005 | 0.009 | 0.002 | 2.96E-09 | 0.022 | TRUE |
| rs436448 | FN BMD | ALM | -0.064 | -0.006 | 0.008 | 0.002 | 1.56E-16 | 0.002 | TRUE |
| rs4448201 | FN BMD | ALM | -0.066 | -0.011 | 0.008 | 0.002 | 4.37E-16 | 0.000 | TRUE |
| rs4759320 | FN BMD | ALM | -0.045 | -0.017 | 0.008 | 0.002 | 3.33E-08 | 0.000 | TRUE |
| rs7108738 | FN BMD | ALM | 0.083 | -0.005 | 0.010 | 0.003 | 8.07E-17 | 0.036 | TRUE |
| rs71390846 | FN BMD | ALM | -0.059 | -0.010 | 0.010 | 0.002 | 3.16E-09 | 0.000 | TRUE |
| rs7209460 | FN BMD | ALM | -0.051 | -0.014 | 0.008 | 0.002 | 1.35E-09 | 0.000 | TRUE |
| rs7524102 | FN BMD | ALM | 0.084 | -0.009 | 0.010 | 0.003 | 7.36E-17 | 0.000 | TRUE |
| rs9478217 | FN BMD | ALM | -0.053 | 0.002 | 0.008 | 0.002 | 1.23E-11 | 0.299 | TRUE |
| rs1023940 | LS BMD | ALM | 0.065 | 0.003 | 0.009 | 0.002 | 6.47E-13 | 0.164 | TRUE |
| rs11002249 | LS BMD | ALM | 0.070 | 0.005 | 0.011 | 0.002 | 1.01E-09 | 0.057 | TRUE |
| rs11024028 | LS BMD | ALM | 0.066 | 0.005 | 0.011 | 0.002 | 1.44E-08 | 0.047 | TRUE |
| rs11680288 | LS BMD | ALM | 0.054 | 0.004 | 0.009 | 0.002 | 3.12E-09 | 0.030 | TRUE |
| rs11692564 | LS BMD | ALM | 0.238 | 0.017 | 0.039 | 0.007 | 4.10E-09 | 0.025 | TRUE |
| rs13046645 | LS BMD | ALM | -0.056 | 0.005 | 0.010 | 0.002 | 2.92E-08 | 0.024 | TRUE |
| rs1357651 | LS BMD | ALM | -0.068 | -0.013 | 0.009 | 0.002 | 3.75E-13 | 0.000 | TRUE |
| rs2220189 | LS BMD | ALM | 0.083 | 0.006 | 0.009 | 0.002 | 4.25E-20 | 0.001 | FALSE |
| rs2235811 | LS BMD | ALM | -0.054 | 0.002 | 0.009 | 0.002 | 4.66E-09 | 0.208 | TRUE |
| rs2291467 | LS BMD | ALM | -0.077 | -0.026 | 0.010 | 0.002 | 9.64E-14 | 0.000 | TRUE |
| rs2566752 | LS BMD | ALM | 0.083 | 0.002 | 0.009 | 0.002 | 1.49E-19 | 0.447 | TRUE |
| rs35681117 | LS BMD | ALM | 0.055 | 0.005 | 0.010 | 0.002 | 2.39E-08 | 0.010 | TRUE |
| rs401680 | LS BMD | ALM | -0.057 | -0.007 | 0.009 | 0.002 | 3.70E-10 | 0.000 | FALSE |
| rs6965122 | LS BMD | ALM | -0.062 | -0.010 | 0.009 | 0.002 | 7.40E-11 | 0.000 | TRUE |
| rs71390846 | LS BMD | ALM | -0.064 | -0.010 | 0.011 | 0.002 | 3.80E-08 | 0.000 | TRUE |
| rs73326583 | LS BMD | ALM | 0.072 | 0.005 | 0.012 | 0.003 | 2.83E-09 | 0.080 | TRUE |
| rs7524102 | LS BMD | ALM | 0.090 | -0.009 | 0.011 | 0.003 | 2.41E-14 | 0.000 | TRUE |
| rs7807953 | LS BMD | ALM | 0.075 | 0.006 | 0.010 | 0.002 | 4.11E-14 | 0.006 | TRUE |
| rs78667121 | LS BMD | ALM | 0.150 | 0.006 | 0.026 | 0.006 | 1.21E-08 | 0.252 | TRUE |
| rs884205 | LS BMD | ALM | -0.062 | -0.003 | 0.010 | 0.002 | 2.77E-09 | 0.252 | TRUE |
| rs894738 | LS BMD | ALM | -0.063 | -0.017 | 0.009 | 0.002 | 2.00E-11 | 0.000 | TRUE |
| rs9533094 | LS BMD | ALM | -0.083 | -0.008 | 0.009 | 0.002 | 2.80E-20 | 0.000 | TRUE |
| rs9749364 | LS BMD | ALM | 0.114 | 0.004 | 0.018 | 0.003 | 6.64E-10 | 0.218 | TRUE |
| rs9921222 | LS BMD | ALM | -0.053 | -0.012 | 0.009 | 0.002 | 3.16E-09 | 0.000 | TRUE |

BMD: bone mineral density; ALM: appendicular lean mass; FA: forearm; FN: femoral neck; LS: lumbar spine; IVs: instrumental variables.
