# Supplementary material for: Osteoporosis and sarcopenia-related traits: A bi-directional Mendelian randomization study
Source: Front Endocrinol (Lausanne). 2022 Sep 14;13:975647. doi: 10.3389/fendo.2022.975647 (PMC9515352; doi:10.3389/fendo.2022.975647)
Supplement: Supplementary file 3 [file Table_3.docx]

Supplementary Table 3. IVs outliers in MR-PRESSO analysis.

| Exposures | Outcomes | IVs selection | No. of IVs | The IVs outliers |
| --- | --- | --- | --- | --- |
| FA BMD | low‐grip strength | All | 15 | NA |
| FA BMD | low‐grip strength | Removed (0) | 15 | NA |
| FA BMD | ALM | All | 16 | rs6894139, rs7216991 |
| FA BMD | ALM | Removed (0) | 16 | rs6894139, rs7216991 |
| FN BMD | low‐grip strength | All | 20 | NA |
| FN BMD | low‐grip strength | Removed (0) | 20 | NA |
| FN BMD | ALM | All | 20 | rs9478217, rs7524102, rs7209460, rs7108738, rs4759320, rs1785493, rs13194508, rs11652763 |
| FN BMD | ALM | Removed (0) | 20 | rs9478217, rs7524102, rs7209460, rs7108738, rs4759320, rs1785493, rs13194508, rs11652763 |
| LS BMD | low‐grip strength | All | 22 | NA |
| LS BMD | low‐grip strength | Removed (0) | 22 | NA |
| LS BMD | ALM | All | 22 | rs9921222, rs894738, rs7524102, rs13046645, rs1357651, rs2235811, rs2291467, rs2566752 |
| LS BMD | ALM | Removed (0) | 22 | rs9921222, rs894738, rs7524102, rs13046645, rs1357651, rs2235811, rs2291467, rs2566752 |
| low‐grip strength | FA BMD | All | 10 | NA |
| low‐grip strength | FA BMD | Removed (1) | 9 | NA |
| low‐grip strength | FN BMD | All | 10 | NA |
| low‐grip strength | FN BMD | Removed (1) | 9 | NA |
| low‐grip strength | LS BMD | All | 10 | NA |
| low‐grip strength | LS BMD | Removed (1) | 9 | NA |
| ALM | FA BMD | All | 562 | rs10040039 |
| ALM | FA BMD | Removed (39) | 523 | rs10040039 |
| ALM | FN BMD | All | 520 | rs10040039, rs2209098, rs6963134, rs7129320, rs894736 |
| ALM | FN BMD | Removed (34) | 486 | rs10040039, rs2209098, rs6963134, rs7129320, rs894736, rs4752689 |
| ALM | LS BMD | All | 519 | rs894736, rs7129320, rs6963134 |
| ALM | LS BMD | Removed (34) | 485 | rs894736, rs7129320, rs6963134 |

Numbers in parentheses depict the number of IVs removed due to confounding.

BMD: bone mineral density; ALM: appendicular lean mass; FA: forearm; FN: femoral neck; LS: lumbar spine; IVs: instrumental variables.
