# Supplementary material for: Osteoporosis and sarcopenia-related traits: A bi-directional Mendelian randomization study
Source: Front Endocrinol (Lausanne). 2022 Sep 14;13:975647. doi: 10.3389/fendo.2022.975647 (PMC9515352; doi:10.3389/fendo.2022.975647)
Supplement: Supplementary file 6 [file Table_6.docx]

Supplementary Table 6. Independent IVs of FA BMD (g/cm3), FN BMD (g/cm3), LS BMD (g/cm3), ALM (kg) and low‐grip strength (kg) in negative control analysis.

| SNP | exposure | outcome | beta.exposure | beta.outcome | se.exposure | se.outcome | pval.exposure | pval.outcome | mr_keep |
| --- | --- | --- | --- | --- | --- | --- | --- | --- | --- |
| rs10952289 | low‐grip strength | myopia | 0.044 | 0.070 | 0.008 | 0.033 | 2.45E-08 | 0.036 | TRUE |
| rs11236213 | low‐grip strength | myopia | -0.050 | 0.047 | 0.008 | 0.036 | 2.98E-10 | 0.193 | TRUE |
| rs13107325 | low‐grip strength | myopia | 0.090 | -0.075 | 0.014 | 0.140 | 8.03E-11 | 0.595 | TRUE |
| rs143384 | low‐grip strength | myopia | 0.055 | 0.043 | 0.008 | 0.033 | 3.68E-13 | 0.192 | TRUE |
| rs143459567 | low‐grip strength | myopia | 0.119 | 0.050 | 0.019 | 0.087 | 3.61E-10 | 0.567 | TRUE |
| rs2899611 | low‐grip strength | myopia | -0.043 | 0.034 | 0.007 | 0.033 | 5.73E-09 | 0.304 | TRUE |
| rs3118903 | low‐grip strength | myopia | 0.058 | -0.005 | 0.009 | 0.037 | 6.40E-11 | 0.899 | TRUE |
| rs34415150 | low‐grip strength | myopia | -0.083 | 0.067 | 0.010 | 0.045 | 3.96E-17 | 0.140 | TRUE |
| rs34464763 | low‐grip strength | myopia | 0.054 | 0.015 | 0.009 | 0.035 | 2.52E-10 | 0.669 | TRUE |
| rs62102286 | low‐grip strength | myopia | 0.049 | -0.069 | 0.007 | 0.036 | 4.67E-11 | 0.052 | TRUE |
| rs7624084 | low‐grip strength | myopia | 0.043 | -0.082 | 0.007 | 0.033 | 7.30E-09 | 0.013 | TRUE |
| rs79723785 | low‐grip strength | myopia | -0.167 | -0.002 | 0.029 | 0.124 | 1.11E-08 | 0.985 | TRUE |
| rs8061064 | low‐grip strength | myopia | 0.041 | 0.026 | 0.007 | 0.033 | 3.80E-08 | 0.431 | FALSE |
| rs958685 | low‐grip strength | myopia | -0.043 | 0.033 | 0.007 | 0.033 | 7.30E-09 | 0.307 | TRUE |
| rs10005035 | ALM | myopia | 0.018 | -0.064 | 0.002 | 0.038 | 7.86E-17 | 0.087 | TRUE |
| rs10008637 | ALM | myopia | 0.013 | -0.027 | 0.002 | 0.033 | 7.80E-12 | 0.412 | TRUE |
| rs10019221 | ALM | myopia | -0.012 | 0.007 | 0.002 | 0.034 | 6.74E-11 | 0.840 | TRUE |
| rs10036789 | ALM | myopia | -0.016 | -0.029 | 0.002 | 0.033 | 9.57E-18 | 0.374 | FALSE |
| rs10040039 | ALM | myopia | 0.016 | 0.029 | 0.002 | 0.036 | 9.57E-18 | 0.416 | TRUE |
| rs10041978 | ALM | myopia | -0.017 | 0.057 | 0.002 | 0.033 | 1.40E-19 | 0.082 | TRUE |
| rs1004982 | ALM | myopia | 0.012 | 0.066 | 0.002 | 0.038 | 4.92E-09 | 0.084 | TRUE |
| rs10058744 | ALM | myopia | 0.015 | -0.014 | 0.002 | 0.033 | 1.54E-14 | 0.676 | TRUE |
| rs10099846 | ALM | myopia | 0.012 | -0.050 | 0.002 | 0.037 | 6.63E-09 | 0.173 | TRUE |
| rs10112506 | ALM | myopia | 0.012 | 0.031 | 0.002 | 0.035 | 2.69E-10 | 0.386 | TRUE |
| rs10119967 | ALM | myopia | -0.035 | -0.037 | 0.002 | 0.043 | 3.66E-53 | 0.397 | TRUE |
| rs10128781 | ALM | myopia | -0.017 | 0.058 | 0.002 | 0.036 | 2.60E-16 | 0.103 | TRUE |
| rs1014526 | ALM | myopia | 0.014 | -0.043 | 0.002 | 0.033 | 5.20E-12 | 0.186 | TRUE |
| rs10170971 | ALM | myopia | -0.016 | -0.003 | 0.002 | 0.033 | 1.42E-16 | 0.933 | FALSE |
| rs10176654 | ALM | myopia | 0.013 | 0.068 | 0.002 | 0.041 | 2.62E-08 | 0.101 | TRUE |
| rs10202701 | ALM | myopia | 0.023 | 0.007 | 0.002 | 0.033 | 6.70E-33 | 0.837 | TRUE |
| rs10208668 | ALM | myopia | 0.042 | -0.204 | 0.005 | 0.121 | 4.04E-16 | 0.092 | TRUE |
| rs10209278 | ALM | myopia | 0.014 | 0.008 | 0.002 | 0.037 | 1.79E-12 | 0.827 | TRUE |
| rs10222594 | ALM | myopia | 0.012 | 0.000 | 0.002 | 0.036 | 8.92E-09 | 0.994 | TRUE |
| rs10241451 | ALM | myopia | -0.015 | -0.051 | 0.002 | 0.041 | 2.89E-10 | 0.213 | TRUE |
| rs10283100 | ALM | myopia | -0.058 | -0.200 | 0.004 | 0.063 | 1.11E-44 | 0.002 | TRUE |
| rs1035583 | ALM | myopia | 0.015 | -0.041 | 0.002 | 0.033 | 6.73E-15 | 0.209 | TRUE |
| rs1040977 | ALM | myopia | -0.024 | 0.008 | 0.003 | 0.051 | 7.99E-22 | 0.882 | TRUE |
| rs10427685 | ALM | myopia | -0.027 | -0.106 | 0.004 | 0.069 | 5.29E-12 | 0.126 | TRUE |
| rs1047891 | ALM | myopia | 0.023 | -0.076 | 0.002 | 0.035 | 2.29E-31 | 0.029 | TRUE |
| rs10483727 | ALM | myopia | 0.037 | -0.100 | 0.002 | 0.036 | 1.43E-83 | 0.005 | TRUE |
| rs10491967 | ALM | myopia | 0.047 | 0.070 | 0.003 | 0.061 | 4.89E-49 | 0.248 | TRUE |
| rs10514518 | ALM | myopia | 0.017 | 0.031 | 0.002 | 0.036 | 1.23E-17 | 0.392 | TRUE |
| rs1051952 | ALM | myopia | -0.013 | 0.011 | 0.002 | 0.033 | 2.56E-12 | 0.735 | TRUE |
| rs1056747 | ALM | myopia | 0.016 | -0.043 | 0.002 | 0.033 | 3.41E-16 | 0.196 | TRUE |
| rs1063582 | ALM | myopia | 0.019 | 0.016 | 0.002 | 0.040 | 4.13E-17 | 0.689 | TRUE |
| rs10748128 | ALM | myopia | 0.026 | -0.037 | 0.002 | 0.034 | 3.12E-37 | 0.284 | TRUE |
| rs10749157 | ALM | myopia | -0.011 | -0.002 | 0.002 | 0.033 | 1.60E-08 | 0.953 | TRUE |
| rs10764692 | ALM | myopia | 0.011 | 0.019 | 0.002 | 0.035 | 2.86E-08 | 0.584 | TRUE |
| rs10776560 | ALM | myopia | -0.016 | 0.068 | 0.002 | 0.034 | 1.42E-16 | 0.046 | TRUE |
| rs10779958 | ALM | myopia | -0.016 | 0.036 | 0.003 | 0.046 | 1.56E-09 | 0.437 | TRUE |
| rs10796828 | ALM | myopia | -0.015 | -0.034 | 0.002 | 0.037 | 1.36E-14 | 0.357 | TRUE |
| rs10807137 | ALM | myopia | -0.046 | -0.001 | 0.003 | 0.046 | 5.16E-74 | 0.979 | TRUE |
| rs10810474 | ALM | myopia | 0.014 | 0.080 | 0.002 | 0.033 | 5.22E-14 | 0.015 | TRUE |
| rs10815304 | ALM | myopia | -0.015 | 0.014 | 0.002 | 0.042 | 5.20E-11 | 0.746 | TRUE |
| rs10822117 | ALM | myopia | 0.018 | 0.001 | 0.002 | 0.037 | 1.24E-15 | 0.987 | TRUE |
| rs10827415 | ALM | myopia | 0.014 | 0.018 | 0.002 | 0.042 | 1.36E-11 | 0.670 | TRUE |
| rs10832963 | ALM | myopia | 0.020 | 0.011 | 0.002 | 0.033 | 2.78E-20 | 0.750 | TRUE |
| rs10840399 | ALM | myopia | -0.021 | 0.043 | 0.003 | 0.049 | 1.56E-11 | 0.376 | TRUE |
| rs10849576 | ALM | myopia | 0.012 | 0.009 | 0.002 | 0.035 | 7.37E-10 | 0.798 | TRUE |
| rs10883555 | ALM | myopia | 0.025 | 0.012 | 0.002 | 0.033 | 9.25E-41 | 0.707 | TRUE |
| rs10922476 | ALM | myopia | -0.016 | -0.025 | 0.002 | 0.035 | 3.73E-17 | 0.470 | TRUE |
| rs1093086 | ALM | myopia | -0.013 | 0.061 | 0.002 | 0.040 | 4.53E-09 | 0.133 | TRUE |
| rs10940169 | ALM | myopia | -0.011 | 0.028 | 0.002 | 0.035 | 7.06E-09 | 0.413 | TRUE |
| rs10995566 | ALM | myopia | -0.012 | -0.038 | 0.002 | 0.035 | 7.75E-10 | 0.281 | TRUE |
| rs11014285 | ALM | myopia | 0.034 | 0.003 | 0.003 | 0.054 | 1.62E-39 | 0.962 | TRUE |
| rs11021305 | ALM | myopia | -0.015 | 0.044 | 0.002 | 0.038 | 1.24E-15 | 0.241 | TRUE |
| rs11042717 | ALM | myopia | 0.029 | -0.019 | 0.002 | 0.033 | 1.35E-52 | 0.557 | TRUE |
| rs11049704 | ALM | myopia | 0.018 | 0.020 | 0.002 | 0.037 | 2.93E-18 | 0.587 | TRUE |
| rs11132166 | ALM | myopia | 0.029 | -0.082 | 0.005 | 0.118 | 5.22E-09 | 0.486 | TRUE |
| rs111365325 | ALM | myopia | -0.027 | -0.005 | 0.002 | 0.041 | 7.23E-35 | 0.901 | TRUE |
| rs11158820 | ALM | myopia | 0.024 | 0.021 | 0.002 | 0.037 | 4.54E-29 | 0.559 | TRUE |
| rs111622870 | ALM | myopia | 0.028 | 0.080 | 0.004 | 0.107 | 1.46E-10 | 0.454 | TRUE |
| rs11175919 | ALM | myopia | 0.035 | 0.042 | 0.006 | 0.099 | 3.31E-09 | 0.669 | TRUE |
| rs11187838 | ALM | myopia | 0.039 | 0.012 | 0.002 | 0.034 | 1.61E-95 | 0.736 | TRUE |
| rs11191208 | ALM | myopia | 0.015 | 0.070 | 0.002 | 0.040 | 9.07E-10 | 0.082 | TRUE |
| rs111925803 | ALM | myopia | 0.012 | 0.027 | 0.002 | 0.037 | 6.27E-09 | 0.466 | TRUE |
| rs11198591 | ALM | myopia | 0.015 | 0.036 | 0.002 | 0.034 | 1.36E-13 | 0.286 | TRUE |
| rs11210229 | ALM | myopia | -0.013 | 0.027 | 0.002 | 0.034 | 4.74E-11 | 0.420 | TRUE |
| rs11233117 | ALM | myopia | 0.018 | 0.005 | 0.002 | 0.033 | 1.98E-20 | 0.873 | FALSE |
| rs112369231 | ALM | myopia | -0.018 | 0.039 | 0.002 | 0.036 | 6.75E-18 | 0.273 | TRUE |
| rs112373502 | ALM | myopia | -0.018 | 0.052 | 0.003 | 0.045 | 1.63E-10 | 0.247 | TRUE |
| rs11243202 | ALM | myopia | -0.030 | 0.037 | 0.002 | 0.033 | 6.89E-57 | 0.254 | TRUE |
| rs112537273 | ALM | myopia | 0.021 | 0.010 | 0.002 | 0.043 | 5.61E-22 | 0.808 | TRUE |
| rs112873218 | ALM | myopia | 0.022 | -0.021 | 0.003 | 0.051 | 3.22E-12 | 0.686 | TRUE |
| rs113146332 | ALM | myopia | 0.031 | -0.217 | 0.005 | 0.152 | 2.20E-10 | 0.153 | TRUE |
| rs113671109 | ALM | myopia | 0.015 | -0.032 | 0.002 | 0.038 | 6.95E-11 | 0.400 | TRUE |
| rs113823725 | ALM | myopia | -0.026 | -0.008 | 0.002 | 0.035 | 2.60E-42 | 0.821 | FALSE |
| rs113827862 | ALM | myopia | 0.024 | 0.092 | 0.004 | 0.062 | 4.23E-09 | 0.137 | TRUE |
| rs113852999 | ALM | myopia | 0.025 | 0.000 | 0.003 | 0.043 | 7.57E-23 | 1.000 | TRUE |
| rs114018835 | ALM | myopia | 0.030 | 0.100 | 0.005 | 0.145 | 2.83E-09 | 0.490 | TRUE |
| rs114192718 | ALM | myopia | -0.022 | 0.073 | 0.004 | 0.078 | 2.34E-09 | 0.350 | TRUE |
| rs114299654 | ALM | myopia | 0.030 | 0.028 | 0.005 | 0.194 | 2.51E-09 | 0.883 | TRUE |
| rs115233595 | ALM | myopia | 0.026 | 0.042 | 0.004 | 0.055 | 1.29E-11 | 0.445 | TRUE |
| rs11590254 | ALM | myopia | -0.019 | -0.015 | 0.002 | 0.034 | 1.40E-20 | 0.666 | TRUE |
| rs115912456 | ALM | myopia | -0.058 | -0.105 | 0.005 | 0.127 | 1.21E-34 | 0.406 | TRUE |
| rs116008080 | ALM | myopia | -0.042 | -0.162 | 0.006 | 0.098 | 4.48E-11 | 0.099 | TRUE |
| rs11605297 | ALM | myopia | 0.015 | -0.039 | 0.002 | 0.037 | 3.22E-11 | 0.295 | TRUE |
| rs11633371 | ALM | myopia | 0.022 | -0.008 | 0.002 | 0.033 | 6.01E-30 | 0.811 | TRUE |
| rs116339650 | ALM | myopia | 0.018 | 0.054 | 0.003 | 0.044 | 1.59E-09 | 0.215 | TRUE |
| rs116493405 | ALM | myopia | 0.029 | 0.005 | 0.004 | 0.099 | 8.30E-12 | 0.963 | TRUE |
| rs11651280 | ALM | myopia | 0.027 | 0.050 | 0.004 | 0.061 | 2.47E-11 | 0.406 | TRUE |
| rs11672848 | ALM | myopia | -0.017 | 0.009 | 0.002 | 0.033 | 2.26E-19 | 0.791 | TRUE |
| rs11684531 | ALM | myopia | 0.017 | 0.091 | 0.003 | 0.064 | 8.11E-10 | 0.154 | TRUE |
| rs117068593 | ALM | myopia | 0.040 | 0.038 | 0.002 | 0.047 | 2.81E-63 | 0.414 | TRUE |
| rs11720869 | ALM | myopia | 0.014 | -0.034 | 0.002 | 0.033 | 1.79E-12 | 0.301 | TRUE |
| rs11721522 | ALM | myopia | -0.011 | 0.065 | 0.002 | 0.035 | 2.42E-08 | 0.062 | TRUE |
| rs11749742 | ALM | myopia | -0.011 | -0.001 | 0.002 | 0.034 | 3.27E-08 | 0.974 | TRUE |
| rs11760961 | ALM | myopia | -0.013 | 0.002 | 0.002 | 0.033 | 2.93E-11 | 0.951 | FALSE |
| rs1177765 | ALM | myopia | 0.023 | 0.016 | 0.002 | 0.033 | 2.73E-34 | 0.631 | TRUE |
| rs11777835 | ALM | myopia | 0.015 | -0.064 | 0.002 | 0.033 | 1.24E-15 | 0.050 | TRUE |
| rs117818446 | ALM | myopia | 0.042 | -0.111 | 0.007 | 0.165 | 4.95E-10 | 0.501 | TRUE |
| rs1190715 | ALM | myopia | -0.010 | -0.003 | 0.002 | 0.033 | 4.41E-08 | 0.932 | TRUE |
| rs11927331 | ALM | myopia | -0.015 | -0.022 | 0.002 | 0.034 | 1.98E-13 | 0.514 | TRUE |
| rs11991823 | ALM | myopia | -0.016 | 0.010 | 0.002 | 0.034 | 8.28E-16 | 0.778 | TRUE |
| rs12037677 | ALM | myopia | 0.018 | 0.005 | 0.002 | 0.036 | 2.33E-17 | 0.897 | TRUE |
| rs12051245 | ALM | myopia | -0.030 | -0.004 | 0.002 | 0.034 | 4.53E-42 | 0.903 | TRUE |
| rs12055045 | ALM | myopia | 0.021 | -0.017 | 0.002 | 0.038 | 2.03E-20 | 0.651 | TRUE |
| rs12099669 | ALM | myopia | 0.033 | 0.047 | 0.002 | 0.034 | 1.60E-61 | 0.176 | TRUE |
| rs1211575 | ALM | myopia | -0.024 | -0.044 | 0.002 | 0.036 | 1.54E-29 | 0.231 | TRUE |
| rs1216743 | ALM | myopia | -0.015 | 0.014 | 0.002 | 0.036 | 4.55E-13 | 0.705 | TRUE |
| rs12185775 | ALM | myopia | -0.017 | 0.055 | 0.003 | 0.048 | 2.60E-08 | 0.247 | TRUE |
| rs12212816 | ALM | myopia | 0.016 | -0.019 | 0.002 | 0.033 | 1.51E-17 | 0.567 | TRUE |
| rs12299065 | ALM | myopia | -0.024 | 0.067 | 0.003 | 0.047 | 1.02E-17 | 0.149 | TRUE |
| rs12334478 | ALM | myopia | 0.016 | 0.037 | 0.002 | 0.033 | 2.38E-17 | 0.266 | FALSE |
| rs12340775 | ALM | myopia | -0.029 | -0.064 | 0.004 | 0.061 | 2.48E-11 | 0.291 | TRUE |
| rs12344515 | ALM | myopia | -0.016 | -0.015 | 0.002 | 0.040 | 1.27E-13 | 0.704 | TRUE |
| rs12347137 | ALM | myopia | 0.046 | -0.030 | 0.002 | 0.039 | 7.03E-82 | 0.442 | TRUE |
| rs12351226 | ALM | myopia | 0.022 | -0.118 | 0.003 | 0.053 | 2.78E-18 | 0.026 | TRUE |
| rs12371664 | ALM | myopia | 0.017 | -0.027 | 0.002 | 0.038 | 1.24E-15 | 0.488 | TRUE |
| rs12463908 | ALM | myopia | 0.017 | -0.003 | 0.002 | 0.041 | 6.20E-12 | 0.935 | TRUE |
| rs12474969 | ALM | myopia | 0.017 | 0.067 | 0.002 | 0.036 | 6.83E-17 | 0.061 | TRUE |
| rs12483401 | ALM | myopia | 0.039 | -0.100 | 0.007 | 0.130 | 7.64E-09 | 0.442 | TRUE |
| rs12509014 | ALM | myopia | -0.026 | 0.017 | 0.002 | 0.043 | 2.80E-30 | 0.686 | TRUE |
| rs12512942 | ALM | myopia | -0.016 | -0.008 | 0.002 | 0.033 | 5.50E-16 | 0.800 | TRUE |
| rs12533452 | ALM | myopia | 0.024 | -0.016 | 0.003 | 0.047 | 7.84E-20 | 0.737 | TRUE |
| rs12595051 | ALM | myopia | 0.018 | -0.005 | 0.002 | 0.036 | 1.54E-17 | 0.898 | TRUE |
| rs1260326 | ALM | myopia | -0.032 | -0.003 | 0.002 | 0.034 | 8.21E-65 | 0.934 | TRUE |
| rs12612857 | ALM | myopia | -0.012 | 0.088 | 0.002 | 0.041 | 1.74E-08 | 0.029 | TRUE |
| rs12622189 | ALM | myopia | 0.027 | 0.006 | 0.002 | 0.034 | 2.28E-38 | 0.870 | TRUE |
| rs12662115 | ALM | myopia | 0.015 | -0.013 | 0.002 | 0.033 | 2.96E-14 | 0.690 | TRUE |
| rs12663031 | ALM | myopia | -0.020 | 0.023 | 0.002 | 0.033 | 1.03E-24 | 0.483 | TRUE |
| rs12679359 | ALM | myopia | -0.026 | 0.020 | 0.003 | 0.048 | 5.84E-21 | 0.680 | TRUE |
| rs12702693 | ALM | myopia | 0.017 | 0.027 | 0.002 | 0.034 | 8.61E-20 | 0.432 | TRUE |
| rs12713004 | ALM | myopia | -0.037 | 0.041 | 0.002 | 0.043 | 2.18E-68 | 0.338 | TRUE |
| rs12714414 | ALM | myopia | 0.035 | 0.108 | 0.003 | 0.044 | 4.63E-39 | 0.014 | TRUE |
| rs12739693 | ALM | myopia | -0.015 | -0.049 | 0.002 | 0.033 | 2.32E-14 | 0.131 | FALSE |
| rs12751807 | ALM | myopia | -0.013 | -0.049 | 0.002 | 0.039 | 4.81E-08 | 0.214 | TRUE |
| rs12761076 | ALM | myopia | -0.026 | 0.007 | 0.002 | 0.037 | 3.31E-35 | 0.853 | TRUE |
| rs12882130 | ALM | myopia | 0.020 | -0.026 | 0.002 | 0.034 | 5.52E-24 | 0.435 | TRUE |
| rs12894822 | ALM | myopia | -0.014 | -0.088 | 0.002 | 0.037 | 8.44E-10 | 0.018 | TRUE |
| rs1290786 | ALM | myopia | -0.014 | 0.062 | 0.002 | 0.033 | 5.22E-14 | 0.061 | TRUE |
| rs12909863 | ALM | myopia | 0.019 | -0.025 | 0.002 | 0.039 | 8.63E-18 | 0.514 | TRUE |
| rs1291114 | ALM | myopia | 0.017 | -0.011 | 0.003 | 0.050 | 2.40E-08 | 0.823 | TRUE |
| rs12997625 | ALM | myopia | -0.017 | -0.002 | 0.002 | 0.033 | 3.64E-19 | 0.944 | TRUE |
| rs13103161 | ALM | myopia | -0.028 | 0.011 | 0.002 | 0.035 | 1.62E-50 | 0.752 | TRUE |
| rs13109280 | ALM | myopia | -0.013 | -0.034 | 0.002 | 0.034 | 5.75E-11 | 0.322 | TRUE |
| rs13112742 | ALM | myopia | -0.015 | -0.011 | 0.003 | 0.048 | 1.54E-09 | 0.818 | TRUE |
| rs13127468 | ALM | myopia | -0.012 | -0.015 | 0.002 | 0.033 | 9.56E-11 | 0.640 | TRUE |
| rs13170063 | ALM | myopia | -0.015 | 0.041 | 0.002 | 0.035 | 1.24E-15 | 0.235 | TRUE |
| rs1317349 | ALM | myopia | -0.026 | -0.009 | 0.002 | 0.035 | 1.08E-34 | 0.790 | TRUE |
| rs1319012 | ALM | myopia | -0.052 | 0.010 | 0.004 | 0.055 | 7.27E-45 | 0.850 | TRUE |
| rs13193017 | ALM | myopia | 0.016 | 0.078 | 0.003 | 0.046 | 1.25E-09 | 0.090 | TRUE |
| rs13209574 | ALM | myopia | -0.029 | -0.057 | 0.003 | 0.059 | 7.17E-20 | 0.336 | TRUE |
| rs13209685 | ALM | myopia | 0.028 | 0.021 | 0.003 | 0.040 | 1.67E-26 | 0.604 | TRUE |
| rs1325596 | ALM | myopia | 0.029 | -0.039 | 0.002 | 0.033 | 1.50E-51 | 0.235 | TRUE |
| rs1330826 | ALM | myopia | 0.016 | 0.042 | 0.002 | 0.038 | 1.87E-12 | 0.261 | TRUE |
| rs13316 | ALM | myopia | 0.012 | 0.003 | 0.002 | 0.033 | 1.42E-09 | 0.939 | TRUE |
| rs13321258 | ALM | myopia | 0.013 | 0.006 | 0.002 | 0.037 | 1.49E-09 | 0.871 | TRUE |
| rs1341215 | ALM | myopia | 0.023 | -0.018 | 0.003 | 0.043 | 2.22E-17 | 0.674 | TRUE |
| rs1355603 | ALM | myopia | -0.047 | 0.069 | 0.003 | 0.044 | 1.52E-77 | 0.116 | TRUE |
| rs139921635 | ALM | myopia | 0.039 | 0.183 | 0.006 | 0.094 | 5.31E-10 | 0.052 | TRUE |
| rs140440099 | ALM | myopia | 0.061 | 0.032 | 0.006 | 0.134 | 2.24E-22 | 0.808 | TRUE |
| rs1405227 | ALM | myopia | 0.013 | 0.012 | 0.002 | 0.034 | 1.12E-10 | 0.721 | TRUE |
| rs141277904 | ALM | myopia | 0.038 | 0.064 | 0.007 | 0.132 | 2.62E-08 | 0.625 | TRUE |
| rs1430157 | ALM | myopia | 0.018 | 0.096 | 0.002 | 0.037 | 9.03E-20 | 0.010 | TRUE |
| rs143076454 | ALM | myopia | -0.050 | 0.045 | 0.007 | 0.141 | 1.01E-12 | 0.747 | TRUE |
| rs143384 | ALM | myopia | -0.073 | 0.043 | 0.002 | 0.033 | 0.00E+00 | 0.192 | TRUE |
| rs1436164 | ALM | myopia | -0.014 | 0.022 | 0.002 | 0.034 | 2.56E-13 | 0.513 | TRUE |
| rs144109601 | ALM | myopia | -0.028 | 0.015 | 0.005 | 0.071 | 6.97E-09 | 0.829 | TRUE |
| rs1444628 | ALM | myopia | 0.024 | 0.077 | 0.002 | 0.040 | 3.55E-33 | 0.053 | TRUE |
| rs1447691 | ALM | myopia | 0.018 | -0.030 | 0.002 | 0.036 | 1.43E-19 | 0.400 | TRUE |
| rs145147649 | ALM | myopia | -0.036 | -0.058 | 0.005 | 0.110 | 8.66E-16 | 0.597 | TRUE |
| rs147110934 | ALM | myopia | -0.072 | 0.183 | 0.006 | 0.220 | 2.43E-31 | 0.407 | TRUE |
| rs1472852 | ALM | myopia | -0.064 | 0.043 | 0.003 | 0.056 | 5.74E-133 | 0.446 | TRUE |
| rs1473441 | ALM | myopia | 0.020 | 0.036 | 0.002 | 0.038 | 4.16E-21 | 0.338 | TRUE |
| rs1487441 | ALM | myopia | 0.014 | -0.034 | 0.002 | 0.033 | 8.19E-13 | 0.301 | TRUE |
| rs149697773 | ALM | myopia | 0.026 | -0.006 | 0.005 | 0.078 | 2.48E-08 | 0.940 | TRUE |
| rs1527149 | ALM | myopia | -0.011 | -0.044 | 0.002 | 0.034 | 2.72E-09 | 0.189 | TRUE |
| rs1557341 | ALM | myopia | -0.015 | -0.029 | 0.002 | 0.037 | 2.01E-14 | 0.430 | TRUE |
| rs1608113 | ALM | myopia | -0.015 | 0.010 | 0.002 | 0.033 | 9.33E-14 | 0.769 | FALSE |
| rs165849 | ALM | myopia | 0.016 | -0.073 | 0.002 | 0.035 | 7.65E-14 | 0.034 | TRUE |
| rs1662842 | ALM | myopia | -0.021 | -0.011 | 0.002 | 0.034 | 1.02E-26 | 0.744 | TRUE |
| rs16844417 | ALM | myopia | 0.023 | -0.016 | 0.003 | 0.048 | 6.54E-15 | 0.742 | TRUE |
| rs17036160 | ALM | myopia | -0.038 | -0.005 | 0.003 | 0.043 | 3.01E-38 | 0.914 | TRUE |
| rs17138358 | ALM | myopia | 0.016 | -0.023 | 0.002 | 0.036 | 1.42E-16 | 0.518 | TRUE |
| rs17197114 | ALM | myopia | -0.018 | 0.042 | 0.003 | 0.045 | 1.44E-12 | 0.349 | TRUE |
| rs17205463 | ALM | myopia | -0.026 | 0.034 | 0.002 | 0.035 | 1.42E-43 | 0.328 | TRUE |
| rs17428810 | ALM | myopia | 0.016 | -0.002 | 0.002 | 0.035 | 9.19E-15 | 0.952 | TRUE |
| rs17478946 | ALM | myopia | 0.019 | 0.027 | 0.002 | 0.037 | 6.08E-20 | 0.467 | TRUE |
| rs17496249 | ALM | myopia | -0.012 | 0.025 | 0.002 | 0.033 | 9.56E-11 | 0.441 | TRUE |
| rs17681189 | ALM | myopia | -0.013 | 0.036 | 0.002 | 0.033 | 5.40E-12 | 0.275 | TRUE |
| rs17713523 | ALM | myopia | 0.011 | -0.056 | 0.002 | 0.033 | 1.97E-09 | 0.088 | TRUE |
| rs177592 | ALM | myopia | -0.022 | 0.030 | 0.003 | 0.060 | 1.28E-12 | 0.612 | TRUE |
| rs17773965 | ALM | myopia | -0.016 | 0.030 | 0.003 | 0.038 | 1.57E-09 | 0.422 | TRUE |
| rs1786263 | ALM | myopia | -0.019 | -0.013 | 0.002 | 0.033 | 1.52E-23 | 0.686 | TRUE |
| rs1800504 | ALM | myopia | -0.013 | 0.022 | 0.002 | 0.033 | 4.74E-11 | 0.511 | TRUE |
| rs1805165 | ALM | myopia | -0.018 | -0.022 | 0.002 | 0.035 | 1.92E-18 | 0.525 | TRUE |
| rs1809179 | ALM | myopia | -0.016 | 0.056 | 0.003 | 0.048 | 2.50E-09 | 0.241 | TRUE |
| rs181766 | ALM | myopia | -0.022 | 0.056 | 0.002 | 0.034 | 2.19E-28 | 0.100 | TRUE |
| rs1899040 | ALM | myopia | 0.015 | -0.051 | 0.002 | 0.040 | 3.88E-11 | 0.208 | TRUE |
| rs1903002 | ALM | myopia | -0.011 | 0.049 | 0.002 | 0.033 | 3.75E-09 | 0.136 | FALSE |
| rs1977337 | ALM | myopia | -0.019 | -0.034 | 0.003 | 0.039 | 8.44E-13 | 0.385 | TRUE |
| rs200439 | ALM | myopia | 0.013 | 0.073 | 0.002 | 0.037 | 2.62E-08 | 0.052 | TRUE |
| rs2007022 | ALM | myopia | 0.018 | 0.014 | 0.002 | 0.046 | 4.07E-16 | 0.767 | TRUE |
| rs201570119 | ALM | myopia | -0.019 | 0.096 | 0.002 | 0.043 | 3.32E-17 | 0.025 | TRUE |
| rs2019203 | ALM | myopia | 0.019 | -0.031 | 0.002 | 0.033 | 2.59E-23 | 0.339 | TRUE |
| rs2035901 | ALM | myopia | -0.024 | -0.043 | 0.002 | 0.033 | 1.41E-36 | 0.195 | TRUE |
| rs2052478 | ALM | myopia | -0.020 | 0.005 | 0.002 | 0.043 | 7.74E-19 | 0.902 | TRUE |
| rs2070598 | ALM | myopia | 0.020 | -0.010 | 0.002 | 0.033 | 6.83E-27 | 0.767 | TRUE |
| rs2071518 | ALM | myopia | -0.024 | -0.045 | 0.002 | 0.041 | 2.65E-29 | 0.269 | TRUE |
| rs2098695 | ALM | myopia | 0.026 | -0.042 | 0.002 | 0.036 | 3.12E-37 | 0.240 | TRUE |
| rs2112617 | ALM | myopia | -0.017 | -0.065 | 0.002 | 0.033 | 1.50E-18 | 0.047 | TRUE |
| rs2125125 | ALM | myopia | -0.016 | -0.048 | 0.002 | 0.045 | 6.44E-12 | 0.288 | TRUE |
| rs212526 | ALM | myopia | -0.021 | 0.012 | 0.002 | 0.034 | 1.99E-29 | 0.712 | TRUE |
| rs2126942 | ALM | myopia | 0.013 | -0.065 | 0.002 | 0.034 | 2.32E-11 | 0.056 | TRUE |
| rs2138374 | ALM | myopia | 0.015 | -0.060 | 0.002 | 0.034 | 9.33E-14 | 0.079 | TRUE |
| rs2140046 | ALM | myopia | 0.019 | -0.065 | 0.002 | 0.033 | 5.24E-24 | 0.048 | TRUE |
| rs2152090 | ALM | myopia | -0.012 | 0.006 | 0.002 | 0.033 | 5.28E-10 | 0.866 | FALSE |
| rs2165772 | ALM | myopia | 0.016 | -0.006 | 0.002 | 0.034 | 1.24E-15 | 0.858 | TRUE |
| rs2174008 | ALM | myopia | -0.019 | 0.036 | 0.002 | 0.033 | 5.24E-24 | 0.275 | FALSE |
| rs2194411 | ALM | myopia | 0.044 | -0.075 | 0.003 | 0.048 | 1.11E-52 | 0.113 | TRUE |
| rs2208404 | ALM | myopia | 0.011 | 0.083 | 0.002 | 0.033 | 5.15E-09 | 0.013 | TRUE |
| rs2209098 | ALM | myopia | -0.024 | 0.006 | 0.002 | 0.036 | 3.55E-33 | 0.865 | TRUE |
| rs2212926 | ALM | myopia | -0.022 | -0.069 | 0.002 | 0.047 | 1.12E-21 | 0.142 | TRUE |
| rs2229840 | ALM | myopia | 0.034 | -0.049 | 0.003 | 0.046 | 2.69E-39 | 0.287 | TRUE |
| rs2240981 | ALM | myopia | 0.013 | 0.001 | 0.002 | 0.038 | 4.81E-08 | 0.982 | TRUE |
| rs2252031 | ALM | myopia | 0.018 | -0.006 | 0.003 | 0.057 | 1.29E-11 | 0.919 | TRUE |
| rs2270894 | ALM | myopia | 0.033 | -0.030 | 0.002 | 0.037 | 1.60E-43 | 0.419 | TRUE |
| rs2280463 | ALM | myopia | 0.015 | -0.017 | 0.002 | 0.038 | 2.56E-12 | 0.661 | TRUE |
| rs2287821 | ALM | myopia | -0.015 | 0.040 | 0.002 | 0.033 | 8.10E-16 | 0.220 | TRUE |
| rs2289976 | ALM | myopia | 0.014 | -0.067 | 0.002 | 0.039 | 6.02E-13 | 0.083 | TRUE |
| rs2296316 | ALM | myopia | 0.019 | 0.047 | 0.002 | 0.033 | 5.24E-24 | 0.151 | TRUE |
| rs2303423 | ALM | myopia | -0.017 | -0.093 | 0.003 | 0.062 | 2.14E-08 | 0.137 | TRUE |
| rs2304655 | ALM | myopia | -0.011 | 0.022 | 0.002 | 0.033 | 2.72E-09 | 0.497 | TRUE |
| rs2305141 | ALM | myopia | -0.018 | -0.008 | 0.002 | 0.037 | 5.88E-22 | 0.819 | TRUE |
| rs2324154 | ALM | myopia | 0.015 | -0.002 | 0.002 | 0.033 | 2.91E-15 | 0.956 | TRUE |
| rs2347808 | ALM | myopia | -0.013 | 0.047 | 0.002 | 0.033 | 4.74E-11 | 0.152 | TRUE |
| rs2436772 | ALM | myopia | 0.022 | 0.035 | 0.002 | 0.043 | 4.81E-22 | 0.417 | TRUE |
| rs244711 | ALM | myopia | 0.028 | -0.031 | 0.002 | 0.035 | 7.46E-37 | 0.374 | TRUE |
| rs2487 | ALM | myopia | -0.014 | 0.042 | 0.002 | 0.037 | 1.73E-13 | 0.249 | TRUE |
| rs2490302 | ALM | myopia | 0.022 | 0.042 | 0.003 | 0.065 | 8.03E-11 | 0.519 | TRUE |
| rs2506697 | ALM | myopia | 0.014 | -0.038 | 0.002 | 0.034 | 1.79E-12 | 0.259 | TRUE |
| rs2524139 | ALM | myopia | -0.042 | -0.053 | 0.002 | 0.038 | 2.76E-99 | 0.163 | TRUE |
| rs2531991 | ALM | myopia | 0.019 | -0.028 | 0.002 | 0.037 | 8.63E-18 | 0.441 | TRUE |
| rs2549677 | ALM | myopia | 0.039 | 0.092 | 0.003 | 0.064 | 1.68E-34 | 0.153 | TRUE |
| rs2569888 | ALM | myopia | 0.013 | 0.037 | 0.002 | 0.038 | 1.49E-09 | 0.322 | TRUE |
| rs2577318 | ALM | myopia | -0.014 | 0.015 | 0.002 | 0.036 | 2.65E-10 | 0.673 | TRUE |
| rs2578565 | ALM | myopia | -0.014 | 0.072 | 0.002 | 0.033 | 1.79E-12 | 0.032 | TRUE |
| rs2582842 | ALM | myopia | -0.012 | -0.017 | 0.002 | 0.037 | 4.91E-08 | 0.650 | TRUE |
| rs2596144 | ALM | myopia | 0.022 | 0.101 | 0.003 | 0.039 | 1.66E-15 | 0.010 | TRUE |
| rs2607234 | ALM | myopia | 0.030 | 0.069 | 0.004 | 0.070 | 2.17E-12 | 0.326 | TRUE |
| rs2609334 | ALM | myopia | 0.017 | -0.004 | 0.002 | 0.035 | 7.68E-15 | 0.918 | TRUE |
| rs2615074 | ALM | myopia | 0.011 | 0.031 | 0.002 | 0.034 | 3.27E-08 | 0.352 | TRUE |
| rs261999 | ALM | myopia | 0.018 | 0.061 | 0.002 | 0.033 | 3.25E-20 | 0.065 | TRUE |
| rs2627702 | ALM | myopia | 0.019 | 0.013 | 0.002 | 0.033 | 8.94E-24 | 0.696 | TRUE |
| rs2648725 | ALM | myopia | 0.017 | -0.002 | 0.002 | 0.043 | 7.29E-13 | 0.969 | TRUE |
| rs2651472 | ALM | myopia | 0.011 | -0.020 | 0.002 | 0.033 | 1.31E-08 | 0.537 | TRUE |
| rs2663126 | ALM | myopia | -0.014 | -0.019 | 0.002 | 0.038 | 3.62E-11 | 0.619 | TRUE |
| rs2678898 | ALM | myopia | 0.013 | 0.033 | 0.002 | 0.034 | 1.13E-11 | 0.332 | TRUE |
| rs2721940 | ALM | myopia | 0.017 | 0.035 | 0.002 | 0.036 | 3.81E-18 | 0.325 | TRUE |
| rs2764264 | ALM | myopia | 0.020 | -0.024 | 0.002 | 0.034 | 4.18E-22 | 0.486 | TRUE |
| rs2788213 | ALM | myopia | 0.012 | -0.014 | 0.002 | 0.037 | 4.71E-09 | 0.692 | TRUE |
| rs2807339 | ALM | myopia | -0.016 | 0.094 | 0.002 | 0.039 | 1.79E-13 | 0.016 | TRUE |
| rs2812208 | ALM | myopia | 0.116 | 0.293 | 0.007 | 0.111 | 1.10E-68 | 0.009 | TRUE |
| rs2823990 | ALM | myopia | -0.013 | 0.018 | 0.002 | 0.034 | 2.15E-10 | 0.595 | TRUE |
| rs2854152 | ALM | myopia | -0.048 | 0.002 | 0.002 | 0.034 | 2.50E-128 | 0.949 | TRUE |
| rs28592876 | ALM | myopia | 0.030 | -0.045 | 0.002 | 0.040 | 6.92E-39 | 0.258 | TRUE |
| rs28678024 | ALM | myopia | 0.012 | -0.028 | 0.002 | 0.035 | 1.46E-08 | 0.417 | TRUE |
| rs28701981 | ALM | myopia | -0.040 | 0.009 | 0.002 | 0.034 | 8.02E-87 | 0.799 | TRUE |
| rs2871865 | ALM | myopia | 0.049 | -0.110 | 0.003 | 0.068 | 1.10E-60 | 0.109 | TRUE |
| rs28736838 | ALM | myopia | -0.012 | 0.042 | 0.002 | 0.035 | 4.92E-09 | 0.230 | TRUE |
| rs28757154 | ALM | myopia | -0.019 | -0.001 | 0.003 | 0.045 | 8.80E-13 | 0.984 | TRUE |
| rs28787734 | ALM | myopia | 0.039 | -0.003 | 0.004 | 0.101 | 5.99E-21 | 0.978 | TRUE |
| rs28817902 | ALM | myopia | -0.023 | 0.007 | 0.003 | 0.066 | 2.17E-15 | 0.921 | TRUE |
| rs2885697 | ALM | myopia | -0.032 | 0.016 | 0.002 | 0.034 | 1.14E-58 | 0.648 | TRUE |
| rs2900208 | ALM | myopia | 0.026 | 0.018 | 0.002 | 0.034 | 3.12E-37 | 0.598 | TRUE |
| rs291970 | ALM | myopia | 0.024 | -0.056 | 0.002 | 0.044 | 1.04E-27 | 0.206 | TRUE |
| rs2925155 | ALM | myopia | -0.015 | -0.036 | 0.002 | 0.037 | 9.22E-12 | 0.327 | TRUE |
| rs2965074 | ALM | myopia | 0.013 | 0.010 | 0.002 | 0.034 | 4.74E-11 | 0.761 | TRUE |
| rs2974337 | ALM | myopia | 0.012 | -0.044 | 0.002 | 0.033 | 7.37E-10 | 0.179 | TRUE |
| rs2993531 | ALM | myopia | -0.018 | -0.011 | 0.002 | 0.034 | 1.98E-20 | 0.750 | TRUE |
| rs2994329 | ALM | myopia | 0.016 | 0.085 | 0.002 | 0.039 | 1.59E-11 | 0.029 | TRUE |
| rs3003137 | ALM | myopia | -0.011 | -0.053 | 0.002 | 0.034 | 1.97E-09 | 0.120 | TRUE |
| rs301805 | ALM | myopia | 0.015 | 0.021 | 0.002 | 0.034 | 1.54E-14 | 0.547 | TRUE |
| rs3103223 | ALM | myopia | -0.013 | -0.024 | 0.002 | 0.039 | 1.02E-08 | 0.539 | TRUE |
| rs3103268 | ALM | myopia | 0.029 | -0.090 | 0.003 | 0.077 | 6.17E-21 | 0.243 | TRUE |
| rs310796 | ALM | myopia | 0.014 | 0.018 | 0.002 | 0.037 | 1.25E-12 | 0.627 | TRUE |
| rs3116602 | ALM | myopia | 0.061 | -0.002 | 0.002 | 0.037 | 5.38E-156 | 0.959 | TRUE |
| rs31196 | ALM | myopia | -0.011 | -0.017 | 0.002 | 0.033 | 1.79E-08 | 0.593 | TRUE |
| rs3184504 | ALM | myopia | -0.018 | -0.062 | 0.002 | 0.033 | 5.88E-22 | 0.060 | TRUE |
| rs332116 | ALM | myopia | -0.021 | -0.087 | 0.002 | 0.033 | 1.02E-22 | 0.009 | TRUE |
| rs33973388 | ALM | myopia | 0.025 | 0.071 | 0.002 | 0.036 | 3.07E-39 | 0.046 | TRUE |
| rs34287 | ALM | myopia | 0.019 | -0.008 | 0.002 | 0.033 | 8.76E-21 | 0.798 | TRUE |
| rs34345560 | ALM | myopia | 0.022 | 0.039 | 0.002 | 0.037 | 7.17E-20 | 0.297 | TRUE |
| rs34517439 | ALM | myopia | 0.042 | -0.042 | 0.003 | 0.049 | 9.42E-48 | 0.391 | TRUE |
| rs34522021 | ALM | myopia | 0.013 | -0.084 | 0.002 | 0.033 | 3.32E-11 | 0.010 | TRUE |
| rs34776209 | ALM | myopia | -0.032 | -0.003 | 0.002 | 0.040 | 4.54E-47 | 0.935 | TRUE |
| rs34786000 | ALM | myopia | -0.015 | 0.065 | 0.002 | 0.033 | 2.91E-15 | 0.048 | TRUE |
| rs35268848 | ALM | myopia | 0.074 | -0.063 | 0.010 | 0.111 | 2.94E-13 | 0.568 | TRUE |
| rs35309034 | ALM | myopia | -0.030 | -0.041 | 0.002 | 0.036 | 1.56E-41 | 0.259 | TRUE |
| rs35453327 | ALM | myopia | 0.028 | 0.001 | 0.004 | 0.098 | 3.81E-15 | 0.991 | TRUE |
| rs35464459 | ALM | myopia | 0.034 | -0.024 | 0.003 | 0.048 | 4.24E-32 | 0.620 | TRUE |
| rs35624335 | ALM | myopia | -0.013 | 0.013 | 0.002 | 0.035 | 1.76E-10 | 0.716 | TRUE |
| rs35696197 | ALM | myopia | 0.013 | 0.091 | 0.002 | 0.043 | 1.47E-09 | 0.036 | TRUE |
| rs35732917 | ALM | myopia | -0.020 | 0.007 | 0.002 | 0.039 | 2.62E-22 | 0.863 | TRUE |
| rs35748083 | ALM | myopia | -0.020 | 0.028 | 0.002 | 0.033 | 3.45E-25 | 0.390 | TRUE |
| rs35756741 | ALM | myopia | -0.038 | -0.080 | 0.003 | 0.054 | 2.23E-30 | 0.138 | TRUE |
| rs35816944 | ALM | myopia | -0.109 | 0.158 | 0.012 | 0.205 | 1.42E-20 | 0.440 | TRUE |
| rs35874463 | ALM | myopia | -0.026 | 0.112 | 0.004 | 0.083 | 1.41E-10 | 0.176 | TRUE |
| rs35892992 | ALM | myopia | -0.018 | 0.072 | 0.002 | 0.042 | 2.51E-15 | 0.087 | TRUE |
| rs35963161 | ALM | myopia | -0.016 | 0.034 | 0.002 | 0.033 | 1.42E-16 | 0.303 | TRUE |
| rs36012032 | ALM | myopia | 0.030 | 0.036 | 0.003 | 0.060 | 1.71E-19 | 0.541 | TRUE |
| rs36048468 | ALM | myopia | 0.025 | 0.027 | 0.002 | 0.045 | 2.36E-28 | 0.544 | TRUE |
| rs36226649 | ALM | myopia | -0.049 | -0.169 | 0.004 | 0.076 | 2.63E-37 | 0.027 | TRUE |
| rs3625 | ALM | myopia | 0.015 | -0.001 | 0.002 | 0.033 | 6.73E-15 | 0.985 | TRUE |
| rs372748371 | ALM | myopia | -0.032 | 0.025 | 0.004 | 0.079 | 5.38E-16 | 0.755 | TRUE |
| rs3742250 | ALM | myopia | 0.014 | -0.058 | 0.002 | 0.033 | 8.19E-13 | 0.075 | FALSE |
| rs3764002 | ALM | myopia | 0.028 | 0.017 | 0.002 | 0.034 | 1.48E-40 | 0.621 | TRUE |
| rs3769885 | ALM | myopia | -0.011 | -0.042 | 0.002 | 0.033 | 5.15E-09 | 0.206 | TRUE |
| rs3792819 | ALM | myopia | -0.021 | 0.053 | 0.003 | 0.091 | 6.56E-10 | 0.560 | TRUE |
| rs3814333 | ALM | myopia | 0.018 | -0.015 | 0.002 | 0.034 | 9.03E-20 | 0.655 | TRUE |
| rs3818416 | ALM | myopia | -0.028 | -0.058 | 0.002 | 0.039 | 7.46E-37 | 0.143 | TRUE |
| rs3830008 | ALM | myopia | 0.015 | -0.049 | 0.002 | 0.037 | 2.15E-11 | 0.193 | TRUE |
| rs3843750 | ALM | myopia | 0.025 | -0.045 | 0.002 | 0.036 | 2.11E-36 | 0.215 | TRUE |
| rs3844 | ALM | myopia | 0.013 | 0.022 | 0.002 | 0.038 | 1.97E-09 | 0.562 | TRUE |
| rs3853252 | ALM | myopia | 0.024 | -0.031 | 0.002 | 0.033 | 7.23E-37 | 0.354 | TRUE |
| rs3901421 | ALM | myopia | 0.022 | 0.040 | 0.002 | 0.033 | 1.10E-29 | 0.216 | FALSE |
| rs395980 | ALM | myopia | 0.018 | 0.027 | 0.002 | 0.037 | 1.92E-18 | 0.466 | TRUE |
| rs4076108 | ALM | myopia | -0.017 | 0.029 | 0.002 | 0.037 | 2.59E-15 | 0.434 | TRUE |
| rs4076427 | ALM | myopia | -0.022 | -0.001 | 0.002 | 0.035 | 1.79E-30 | 0.979 | TRUE |
| rs4121583 | ALM | myopia | 0.012 | -0.042 | 0.002 | 0.034 | 3.64E-09 | 0.210 | TRUE |
| rs41271299 | ALM | myopia | 0.062 | -0.067 | 0.004 | 0.130 | 1.51E-46 | 0.608 | TRUE |
| rs41298373 | ALM | myopia | 0.019 | 0.002 | 0.003 | 0.048 | 2.41E-09 | 0.965 | TRUE |
| rs41311445 | ALM | myopia | 0.033 | -0.012 | 0.003 | 0.048 | 1.18E-24 | 0.796 | TRUE |
| rs42039 | ALM | myopia | 0.048 | -0.017 | 0.002 | 0.039 | 5.77E-106 | 0.665 | TRUE |
| rs4244809 | ALM | myopia | -0.026 | -0.037 | 0.002 | 0.043 | 4.62E-30 | 0.384 | TRUE |
| rs4252548 | ALM | myopia | -0.075 | -0.019 | 0.007 | 0.102 | 4.93E-31 | 0.853 | TRUE |
| rs4274112 | ALM | myopia | 0.022 | -0.022 | 0.002 | 0.034 | 1.99E-27 | 0.527 | TRUE |
| rs4282339 | ALM | myopia | -0.031 | 0.024 | 0.002 | 0.042 | 1.16E-41 | 0.562 | TRUE |
| rs4287835 | ALM | myopia | -0.015 | -0.018 | 0.002 | 0.033 | 1.02E-14 | 0.583 | TRUE |
| rs4360494 | ALM | myopia | -0.020 | 0.010 | 0.002 | 0.033 | 1.99E-25 | 0.773 | FALSE |
| rs4383083 | ALM | myopia | 0.011 | -0.028 | 0.002 | 0.037 | 2.86E-08 | 0.442 | TRUE |
| rs4444637 | ALM | myopia | -0.018 | 0.014 | 0.003 | 0.047 | 3.37E-11 | 0.770 | TRUE |
| rs4472895 | ALM | myopia | -0.015 | 0.000 | 0.002 | 0.041 | 1.73E-11 | 0.999 | TRUE |
| rs447352 | ALM | myopia | -0.018 | 0.039 | 0.003 | 0.055 | 4.34E-10 | 0.477 | TRUE |
| rs4504126 | ALM | myopia | -0.046 | 0.046 | 0.006 | 0.133 | 2.17E-15 | 0.729 | TRUE |
| rs45474992 | ALM | myopia | -0.062 | -0.030 | 0.005 | 0.088 | 1.08E-33 | 0.737 | TRUE |
| rs45528934 | ALM | myopia | 0.026 | -0.027 | 0.003 | 0.048 | 6.99E-24 | 0.574 | TRUE |
| rs4554207 | ALM | myopia | 0.011 | 0.030 | 0.002 | 0.033 | 1.97E-09 | 0.364 | TRUE |
| rs4615815 | ALM | myopia | 0.025 | 0.049 | 0.002 | 0.035 | 2.43E-38 | 0.158 | TRUE |
| rs4619294 | ALM | myopia | -0.014 | 0.026 | 0.002 | 0.036 | 7.03E-12 | 0.470 | TRUE |
| rs4622329 | ALM | myopia | 0.015 | 0.044 | 0.002 | 0.033 | 9.33E-14 | 0.187 | TRUE |
| rs4648620 | ALM | myopia | -0.013 | 0.017 | 0.002 | 0.033 | 4.74E-11 | 0.606 | FALSE |
| rs4652902 | ALM | myopia | 0.013 | -0.038 | 0.002 | 0.036 | 4.53E-09 | 0.298 | TRUE |
| rs465983 | ALM | myopia | 0.015 | 0.012 | 0.002 | 0.036 | 3.54E-12 | 0.741 | TRUE |
| rs4682483 | ALM | myopia | -0.017 | 0.034 | 0.003 | 0.037 | 2.21E-10 | 0.351 | TRUE |
| rs4733775 | ALM | myopia | 0.014 | 0.002 | 0.002 | 0.034 | 8.68E-13 | 0.942 | TRUE |
| rs4735761 | ALM | myopia | -0.033 | -0.044 | 0.002 | 0.039 | 5.69E-56 | 0.261 | TRUE |
| rs4752689 | ALM | myopia | 0.021 | -0.031 | 0.002 | 0.036 | 3.86E-27 | 0.386 | TRUE |
| rs4752829 | ALM | myopia | 0.026 | -0.053 | 0.002 | 0.034 | 1.01E-35 | 0.112 | TRUE |
| rs4754296 | ALM | myopia | 0.017 | 0.013 | 0.003 | 0.039 | 6.20E-10 | 0.732 | TRUE |
| rs4763327 | ALM | myopia | 0.012 | -0.024 | 0.002 | 0.033 | 4.71E-09 | 0.477 | TRUE |
| rs4776624 | ALM | myopia | -0.015 | -0.023 | 0.002 | 0.035 | 2.91E-15 | 0.505 | TRUE |
| rs4799799 | ALM | myopia | -0.011 | 0.012 | 0.002 | 0.033 | 1.60E-08 | 0.711 | TRUE |
| rs4815952 | ALM | myopia | 0.016 | 0.015 | 0.002 | 0.033 | 2.38E-17 | 0.643 | TRUE |
| rs4847378 | ALM | myopia | 0.014 | -0.023 | 0.002 | 0.033 | 8.19E-13 | 0.497 | TRUE |
| rs4849904 | ALM | myopia | -0.011 | 0.031 | 0.002 | 0.037 | 5.15E-09 | 0.390 | TRUE |
| rs4852257 | ALM | myopia | 0.023 | -0.080 | 0.002 | 0.033 | 5.21E-34 | 0.015 | TRUE |
| rs485554 | ALM | myopia | 0.034 | 0.030 | 0.002 | 0.035 | 1.48E-65 | 0.388 | TRUE |
| rs4895801 | ALM | myopia | -0.015 | -0.088 | 0.002 | 0.033 | 4.43E-15 | 0.007 | FALSE |
| rs4909912 | ALM | myopia | -0.028 | -0.007 | 0.002 | 0.033 | 3.82E-48 | 0.839 | TRUE |
| rs4934377 | ALM | myopia | -0.017 | 0.050 | 0.002 | 0.035 | 2.60E-16 | 0.159 | TRUE |
| rs4940874 | ALM | myopia | -0.015 | 0.061 | 0.002 | 0.039 | 6.97E-10 | 0.115 | TRUE |
| rs496783 | ALM | myopia | 0.012 | 0.127 | 0.002 | 0.034 | 6.74E-11 | 0.000 | TRUE |
| rs4976262 | ALM | myopia | 0.025 | -0.006 | 0.002 | 0.034 | 1.68E-34 | 0.854 | TRUE |
| rs4985445 | ALM | myopia | 0.018 | 0.009 | 0.002 | 0.033 | 3.25E-20 | 0.777 | TRUE |
| rs501250 | ALM | myopia | -0.013 | 0.036 | 0.002 | 0.033 | 7.80E-12 | 0.278 | TRUE |
| rs501811 | ALM | myopia | 0.020 | -0.009 | 0.003 | 0.043 | 3.28E-11 | 0.833 | TRUE |
| rs55633823 | ALM | myopia | 0.015 | 0.032 | 0.002 | 0.041 | 3.22E-11 | 0.436 | TRUE |
| rs55745410 | ALM | myopia | 0.016 | 0.079 | 0.002 | 0.034 | 4.16E-15 | 0.020 | TRUE |
| rs55758152 | ALM | myopia | 0.015 | -0.002 | 0.002 | 0.034 | 4.17E-13 | 0.961 | TRUE |
| rs55872725 | ALM | myopia | 0.022 | 0.035 | 0.002 | 0.033 | 1.53E-31 | 0.284 | TRUE |
| rs55877758 | ALM | myopia | -0.039 | -0.077 | 0.002 | 0.042 | 1.00E-71 | 0.065 | TRUE |
| rs56207248 | ALM | myopia | -0.027 | 0.038 | 0.004 | 0.069 | 6.76E-13 | 0.581 | TRUE |
| rs56207600 | ALM | myopia | 0.019 | 0.070 | 0.003 | 0.048 | 1.55E-10 | 0.145 | TRUE |
| rs56208656 | ALM | myopia | -0.028 | -0.010 | 0.003 | 0.051 | 6.66E-17 | 0.849 | TRUE |
| rs56263064 | ALM | myopia | 0.015 | -0.037 | 0.002 | 0.039 | 9.14E-13 | 0.334 | TRUE |
| rs56309431 | ALM | myopia | 0.017 | 0.024 | 0.003 | 0.053 | 8.48E-09 | 0.651 | TRUE |
| rs56363908 | ALM | myopia | 0.038 | -0.047 | 0.005 | 0.090 | 4.38E-16 | 0.601 | TRUE |
| rs5742915 | ALM | myopia | -0.025 | -0.013 | 0.002 | 0.033 | 6.14E-39 | 0.692 | TRUE |
| rs577289 | ALM | myopia | 0.013 | -0.068 | 0.002 | 0.036 | 2.64E-09 | 0.056 | TRUE |
| rs57904377 | ALM | myopia | 0.018 | 0.045 | 0.002 | 0.045 | 1.64E-13 | 0.321 | TRUE |
| rs58738817 | ALM | myopia | -0.026 | -0.023 | 0.002 | 0.036 | 1.58E-31 | 0.513 | TRUE |
| rs59000092 | ALM | myopia | 0.020 | 0.034 | 0.002 | 0.037 | 1.03E-20 | 0.356 | TRUE |
| rs591668 | ALM | myopia | -0.017 | 0.057 | 0.002 | 0.033 | 5.29E-20 | 0.088 | TRUE |
| rs59753424 | ALM | myopia | 0.020 | 0.002 | 0.002 | 0.041 | 9.82E-20 | 0.971 | TRUE |
| rs59950280 | ALM | myopia | -0.025 | 0.041 | 0.002 | 0.038 | 5.91E-37 | 0.283 | TRUE |
| rs59951000 | ALM | myopia | -0.040 | 0.018 | 0.005 | 0.060 | 1.73E-17 | 0.760 | TRUE |
| rs59985551 | ALM | myopia | -0.031 | 0.035 | 0.002 | 0.039 | 6.21E-46 | 0.368 | TRUE |
| rs6000890 | ALM | myopia | 0.014 | 0.013 | 0.002 | 0.034 | 1.48E-11 | 0.698 | TRUE |
| rs6028716 | ALM | myopia | -0.021 | -0.001 | 0.002 | 0.042 | 1.36E-21 | 0.984 | TRUE |
| rs60328144 | ALM | myopia | 0.014 | 0.004 | 0.002 | 0.033 | 3.48E-14 | 0.903 | TRUE |
| rs603486 | ALM | myopia | -0.013 | 0.014 | 0.002 | 0.034 | 2.15E-10 | 0.673 | TRUE |
| rs60408354 | ALM | myopia | 0.026 | -0.033 | 0.004 | 0.060 | 6.27E-13 | 0.583 | TRUE |
| rs6054390 | ALM | myopia | -0.019 | -0.001 | 0.002 | 0.036 | 5.46E-21 | 0.980 | TRUE |
| rs6054491 | ALM | myopia | 0.014 | 0.014 | 0.002 | 0.037 | 1.09E-10 | 0.703 | TRUE |
| rs6066122 | ALM | myopia | -0.013 | -0.014 | 0.002 | 0.045 | 3.36E-08 | 0.751 | TRUE |
| rs60804050 | ALM | myopia | -0.022 | 0.043 | 0.002 | 0.038 | 4.98E-25 | 0.262 | TRUE |
| rs6082354 | ALM | myopia | 0.024 | -0.032 | 0.002 | 0.036 | 3.55E-33 | 0.378 | TRUE |
| rs612577 | ALM | myopia | 0.015 | -0.042 | 0.003 | 0.042 | 5.03E-09 | 0.319 | TRUE |
| rs61729527 | ALM | myopia | -0.035 | 0.049 | 0.004 | 0.066 | 8.52E-16 | 0.460 | TRUE |
| rs61732778 | ALM | myopia | 0.023 | 0.004 | 0.004 | 0.059 | 5.09E-10 | 0.943 | TRUE |
| rs61838776 | ALM | myopia | 0.031 | 0.003 | 0.003 | 0.049 | 1.59E-35 | 0.950 | TRUE |
| rs61925210 | ALM | myopia | -0.019 | -0.048 | 0.003 | 0.051 | 1.62E-09 | 0.342 | TRUE |
| rs61940146 | ALM | myopia | -0.011 | -0.012 | 0.002 | 0.034 | 2.14E-08 | 0.722 | TRUE |
| rs61944841 | ALM | myopia | 0.025 | 0.042 | 0.002 | 0.034 | 1.12E-36 | 0.215 | TRUE |
| rs62033029 | ALM | myopia | -0.014 | 0.062 | 0.002 | 0.043 | 8.76E-10 | 0.147 | TRUE |
| rs62048221 | ALM | myopia | -0.024 | -0.040 | 0.002 | 0.041 | 3.82E-28 | 0.334 | TRUE |
| rs62103240 | ALM | myopia | 0.021 | -0.012 | 0.004 | 0.075 | 1.01E-08 | 0.879 | TRUE |
| rs62143873 | ALM | myopia | -0.012 | -0.019 | 0.002 | 0.033 | 1.42E-09 | 0.578 | TRUE |
| rs62177315 | ALM | myopia | 0.018 | 0.026 | 0.003 | 0.066 | 3.18E-08 | 0.696 | TRUE |
| rs62372061 | ALM | myopia | 0.039 | -0.054 | 0.003 | 0.050 | 7.76E-35 | 0.285 | TRUE |
| rs62449290 | ALM | myopia | 0.021 | 0.029 | 0.003 | 0.047 | 2.56E-16 | 0.541 | TRUE |
| rs62466110 | ALM | myopia | 0.037 | 0.059 | 0.004 | 0.048 | 1.45E-19 | 0.223 | TRUE |
| rs62501195 | ALM | myopia | 0.020 | 0.042 | 0.003 | 0.039 | 2.38E-15 | 0.274 | TRUE |
| rs62621812 | ALM | myopia | 0.074 | 0.072 | 0.007 | 0.081 | 4.87E-27 | 0.377 | TRUE |
| rs6444847 | ALM | myopia | -0.011 | -0.055 | 0.002 | 0.035 | 3.80E-08 | 0.116 | TRUE |
| rs6450136 | ALM | myopia | -0.020 | 0.026 | 0.002 | 0.038 | 9.19E-24 | 0.487 | TRUE |
| rs6450961 | ALM | myopia | 0.012 | -0.042 | 0.002 | 0.034 | 3.64E-09 | 0.217 | TRUE |
| rs6452875 | ALM | myopia | 0.013 | -0.035 | 0.002 | 0.040 | 4.81E-08 | 0.383 | TRUE |
| rs6461948 | ALM | myopia | 0.011 | -0.008 | 0.002 | 0.037 | 2.14E-08 | 0.823 | TRUE |
| rs6469845 | ALM | myopia | 0.014 | 0.021 | 0.002 | 0.036 | 8.44E-10 | 0.554 | TRUE |
| rs6501381 | ALM | myopia | 0.034 | -0.035 | 0.003 | 0.057 | 6.13E-30 | 0.537 | TRUE |
| rs6543146 | ALM | myopia | -0.015 | -0.069 | 0.002 | 0.037 | 5.26E-16 | 0.059 | TRUE |
| rs6544743 | ALM | myopia | 0.022 | 0.041 | 0.002 | 0.038 | 1.54E-19 | 0.280 | TRUE |
| rs655113 | ALM | myopia | -0.019 | -0.037 | 0.002 | 0.035 | 3.48E-19 | 0.281 | TRUE |
| rs6567160 | ALM | myopia | -0.049 | -0.043 | 0.002 | 0.042 | 5.15E-109 | 0.307 | TRUE |
| rs6570509 | ALM | myopia | -0.024 | -0.018 | 0.002 | 0.037 | 3.30E-31 | 0.632 | TRUE |
| rs66613683 | ALM | myopia | -0.015 | -0.032 | 0.003 | 0.047 | 1.97E-09 | 0.493 | TRUE |
| rs6693481 | ALM | myopia | 0.014 | 0.002 | 0.002 | 0.034 | 8.68E-13 | 0.961 | TRUE |
| rs670129 | ALM | myopia | -0.012 | 0.008 | 0.002 | 0.035 | 5.28E-10 | 0.816 | TRUE |
| rs670318 | ALM | myopia | -0.041 | -0.059 | 0.004 | 0.070 | 6.21E-21 | 0.395 | TRUE |
| rs6738207 | ALM | myopia | 0.013 | -0.024 | 0.002 | 0.035 | 2.32E-11 | 0.493 | TRUE |
| rs6739394 | ALM | myopia | -0.014 | 0.017 | 0.002 | 0.033 | 3.48E-14 | 0.614 | TRUE |
| rs6762851 | ALM | myopia | 0.022 | 0.004 | 0.002 | 0.033 | 1.15E-27 | 0.908 | TRUE |
| rs68083605 | ALM | myopia | 0.019 | -0.036 | 0.002 | 0.035 | 2.59E-23 | 0.308 | TRUE |
| rs680882 | ALM | myopia | -0.013 | -0.034 | 0.002 | 0.037 | 1.49E-09 | 0.365 | TRUE |
| rs6844176 | ALM | myopia | -0.013 | -0.045 | 0.002 | 0.033 | 1.13E-11 | 0.176 | TRUE |
| rs6852065 | ALM | myopia | 0.013 | -0.026 | 0.002 | 0.034 | 5.40E-12 | 0.446 | TRUE |
| rs6854705 | ALM | myopia | 0.017 | 0.021 | 0.002 | 0.042 | 5.66E-13 | 0.613 | TRUE |
| rs6860245 | ALM | myopia | 0.059 | 0.020 | 0.002 | 0.045 | 6.72E-158 | 0.657 | TRUE |
| rs6874142 | ALM | myopia | -0.029 | -0.033 | 0.003 | 0.057 | 1.54E-20 | 0.557 | TRUE |
| rs6899155 | ALM | myopia | 0.028 | -0.040 | 0.002 | 0.033 | 7.83E-50 | 0.234 | TRUE |
| rs6910414 | ALM | myopia | 0.014 | -0.002 | 0.002 | 0.041 | 3.29E-09 | 0.964 | TRUE |
| rs6919321 | ALM | myopia | 0.019 | 0.054 | 0.002 | 0.036 | 5.24E-24 | 0.138 | TRUE |
| rs6923230 | ALM | myopia | 0.013 | -0.089 | 0.002 | 0.033 | 2.32E-11 | 0.007 | TRUE |
| rs6931421 | ALM | myopia | 0.028 | 0.014 | 0.002 | 0.036 | 3.15E-44 | 0.696 | TRUE |
| rs6943386 | ALM | myopia | -0.011 | 0.004 | 0.002 | 0.033 | 2.42E-08 | 0.904 | TRUE |
| rs6960741 | ALM | myopia | -0.019 | -0.021 | 0.003 | 0.045 | 1.78E-10 | 0.640 | TRUE |
| rs6962887 | ALM | myopia | 0.013 | 0.017 | 0.002 | 0.034 | 1.47E-09 | 0.612 | TRUE |
| rs6963134 | ALM | myopia | -0.014 | -0.002 | 0.002 | 0.037 | 7.38E-12 | 0.954 | TRUE |
| rs6975015 | ALM | myopia | 0.021 | 0.039 | 0.003 | 0.056 | 3.44E-13 | 0.484 | TRUE |
| rs6977416 | ALM | myopia | 0.046 | -0.060 | 0.002 | 0.034 | 1.46E-115 | 0.077 | TRUE |
| rs700677 | ALM | myopia | 0.017 | -0.022 | 0.002 | 0.036 | 5.15E-18 | 0.547 | TRUE |
| rs7014590 | ALM | myopia | 0.023 | 0.052 | 0.002 | 0.038 | 3.63E-25 | 0.167 | TRUE |
| rs704660 | ALM | myopia | 0.015 | -0.040 | 0.002 | 0.033 | 8.10E-16 | 0.230 | TRUE |
| rs704832 | ALM | myopia | -0.012 | 0.002 | 0.002 | 0.040 | 4.91E-08 | 0.964 | TRUE |
| rs705953 | ALM | myopia | 0.019 | 0.039 | 0.002 | 0.034 | 2.10E-21 | 0.248 | TRUE |
| rs7078507 | ALM | myopia | 0.020 | -0.038 | 0.002 | 0.034 | 6.53E-26 | 0.261 | TRUE |
| rs7083556 | ALM | myopia | -0.011 | -0.027 | 0.002 | 0.033 | 9.65E-09 | 0.417 | FALSE |
| rs7095087 | ALM | myopia | 0.012 | -0.056 | 0.002 | 0.037 | 4.92E-09 | 0.129 | TRUE |
| rs7095472 | ALM | myopia | -0.027 | 0.041 | 0.002 | 0.033 | 7.42E-45 | 0.207 | TRUE |
| rs7107356 | ALM | myopia | -0.013 | 0.015 | 0.002 | 0.033 | 2.56E-12 | 0.659 | TRUE |
| rs7129320 | ALM | myopia | -0.039 | 0.079 | 0.003 | 0.058 | 1.36E-54 | 0.174 | TRUE |
| rs7136054 | ALM | myopia | -0.050 | -0.018 | 0.002 | 0.033 | 8.01E-151 | 0.587 | FALSE |
| rs7137546 | ALM | myopia | -0.014 | 0.014 | 0.002 | 0.033 | 7.80E-14 | 0.674 | FALSE |
| rs71519447 | ALM | myopia | -0.072 | 0.055 | 0.003 | 0.052 | 6.80E-126 | 0.291 | TRUE |
| rs7164187 | ALM | myopia | 0.015 | 0.072 | 0.002 | 0.034 | 1.91E-15 | 0.034 | TRUE |
| rs7171129 | ALM | myopia | 0.012 | -0.014 | 0.002 | 0.037 | 1.97E-09 | 0.710 | TRUE |
| rs7185244 | ALM | myopia | 0.015 | 0.017 | 0.002 | 0.042 | 1.24E-10 | 0.684 | TRUE |
| rs718603 | ALM | myopia | 0.013 | 0.031 | 0.002 | 0.037 | 4.43E-10 | 0.404 | TRUE |
| rs7225068 | ALM | myopia | -0.014 | -0.024 | 0.002 | 0.033 | 1.16E-13 | 0.471 | TRUE |
| rs7228151 | ALM | myopia | 0.019 | 0.012 | 0.002 | 0.037 | 8.73E-16 | 0.740 | TRUE |
| rs7229520 | ALM | myopia | -0.022 | -0.008 | 0.002 | 0.034 | 4.08E-29 | 0.820 | TRUE |
| rs723149 | ALM | myopia | 0.028 | -0.008 | 0.002 | 0.033 | 8.25E-48 | 0.807 | TRUE |
| rs7259285 | ALM | myopia | -0.013 | 0.052 | 0.002 | 0.033 | 3.72E-12 | 0.111 | TRUE |
| rs72771070 | ALM | myopia | 0.015 | -0.056 | 0.002 | 0.035 | 9.14E-13 | 0.107 | TRUE |
| rs72801818 | ALM | myopia | 0.031 | -0.027 | 0.002 | 0.037 | 3.07E-50 | 0.478 | TRUE |
| rs72809820 | ALM | myopia | -0.011 | 0.003 | 0.002 | 0.033 | 2.86E-08 | 0.916 | TRUE |
| rs72829852 | ALM | myopia | 0.031 | 0.086 | 0.004 | 0.065 | 2.32E-15 | 0.187 | TRUE |
| rs72841270 | ALM | myopia | -0.029 | -0.122 | 0.003 | 0.047 | 8.64E-26 | 0.009 | TRUE |
| rs7286917 | ALM | myopia | -0.017 | -0.043 | 0.002 | 0.037 | 1.05E-13 | 0.245 | TRUE |
| rs72908840 | ALM | myopia | 0.037 | -0.067 | 0.004 | 0.088 | 7.65E-18 | 0.444 | TRUE |
| rs7301341 | ALM | myopia | 0.026 | -0.043 | 0.002 | 0.034 | 3.12E-37 | 0.216 | TRUE |
| rs73040028 | ALM | myopia | 0.017 | 0.040 | 0.002 | 0.037 | 2.23E-14 | 0.284 | TRUE |
| rs73125634 | ALM | myopia | -0.020 | 0.048 | 0.002 | 0.034 | 1.61E-20 | 0.162 | TRUE |
| rs73186333 | ALM | myopia | -0.037 | 0.024 | 0.006 | 0.095 | 3.21E-09 | 0.800 | TRUE |
| rs7320878 | ALM | myopia | -0.015 | 0.008 | 0.002 | 0.033 | 2.91E-15 | 0.806 | TRUE |
| rs7328187 | ALM | myopia | -0.012 | -0.012 | 0.002 | 0.033 | 1.03E-09 | 0.709 | TRUE |
| rs73382475 | ALM | myopia | 0.025 | -0.074 | 0.003 | 0.059 | 1.39E-14 | 0.207 | TRUE |
| rs73384223 | ALM | myopia | 0.021 | 0.027 | 0.002 | 0.042 | 1.32E-17 | 0.516 | TRUE |
| rs73490624 | ALM | myopia | -0.018 | 0.085 | 0.002 | 0.041 | 1.77E-14 | 0.040 | TRUE |
| rs7359097 | ALM | myopia | 0.012 | 0.025 | 0.002 | 0.033 | 1.91E-10 | 0.456 | TRUE |
| rs7367519 | ALM | myopia | -0.016 | -0.002 | 0.002 | 0.040 | 2.40E-16 | 0.970 | TRUE |
| rs74048171 | ALM | myopia | -0.012 | -0.015 | 0.002 | 0.039 | 3.80E-08 | 0.695 | TRUE |
| rs7418410 | ALM | myopia | 0.016 | -0.021 | 0.002 | 0.034 | 3.41E-16 | 0.534 | TRUE |
| rs7428883 | ALM | myopia | -0.028 | -0.026 | 0.002 | 0.044 | 2.10E-33 | 0.555 | TRUE |
| rs7485647 | ALM | myopia | -0.026 | 0.025 | 0.003 | 0.037 | 1.03E-23 | 0.498 | TRUE |
| rs75022676 | ALM | myopia | -0.016 | 0.072 | 0.002 | 0.037 | 1.37E-12 | 0.052 | TRUE |
| rs7512641 | ALM | myopia | -0.019 | 0.006 | 0.002 | 0.035 | 4.13E-17 | 0.867 | TRUE |
| rs75172776 | ALM | myopia | -0.023 | 0.093 | 0.004 | 0.080 | 1.03E-09 | 0.245 | TRUE |
| rs75478182 | ALM | myopia | 0.023 | -0.027 | 0.003 | 0.066 | 3.18E-12 | 0.680 | TRUE |
| rs75702986 | ALM | myopia | -0.016 | -0.064 | 0.003 | 0.040 | 7.03E-11 | 0.114 | TRUE |
| rs757042 | ALM | myopia | 0.014 | 0.002 | 0.002 | 0.035 | 2.62E-11 | 0.955 | TRUE |
| rs7574162 | ALM | myopia | 0.015 | -0.030 | 0.002 | 0.039 | 3.54E-12 | 0.442 | TRUE |
| rs7582516 | ALM | myopia | 0.025 | 0.023 | 0.002 | 0.037 | 5.91E-37 | 0.526 | TRUE |
| rs7598430 | ALM | myopia | -0.016 | -0.025 | 0.002 | 0.033 | 3.73E-17 | 0.449 | TRUE |
| rs7633464 | ALM | myopia | 0.018 | 0.031 | 0.002 | 0.033 | 3.25E-20 | 0.346 | TRUE |
| rs76364830 | ALM | myopia | -0.047 | -0.026 | 0.004 | 0.089 | 1.40E-33 | 0.774 | TRUE |
| rs7646501 | ALM | myopia | 0.017 | 0.006 | 0.002 | 0.036 | 5.72E-16 | 0.864 | TRUE |
| rs76488803 | ALM | myopia | -0.024 | -0.008 | 0.003 | 0.074 | 1.68E-12 | 0.918 | TRUE |
| rs76520574 | ALM | myopia | -0.045 | 0.052 | 0.005 | 0.078 | 5.04E-20 | 0.504 | TRUE |
| rs7666804 | ALM | myopia | 0.017 | 0.002 | 0.002 | 0.034 | 1.50E-18 | 0.943 | TRUE |
| rs76693355 | ALM | myopia | 0.027 | 0.052 | 0.003 | 0.049 | 4.13E-19 | 0.294 | TRUE |
| rs76895963 | ALM | myopia | -0.164 | 0.103 | 0.007 | 0.098 | 1.22E-111 | 0.293 | TRUE |
| rs7692387 | ALM | myopia | 0.017 | -0.032 | 0.002 | 0.038 | 1.90E-12 | 0.406 | TRUE |
| rs7701233 | ALM | myopia | 0.018 | 0.001 | 0.002 | 0.033 | 4.47E-21 | 0.984 | TRUE |
| rs7731023 | ALM | myopia | -0.017 | 0.008 | 0.002 | 0.034 | 2.40E-18 | 0.807 | TRUE |
| rs77542162 | ALM | myopia | -0.058 | 0.171 | 0.006 | 0.201 | 2.26E-19 | 0.396 | TRUE |
| rs7761910 | ALM | myopia | -0.016 | 0.009 | 0.002 | 0.035 | 1.24E-15 | 0.796 | TRUE |
| rs7768382 | ALM | myopia | 0.020 | -0.020 | 0.002 | 0.033 | 3.73E-26 | 0.534 | TRUE |
| rs7768973 | ALM | myopia | -0.024 | 0.000 | 0.002 | 0.033 | 1.41E-36 | 0.999 | FALSE |
| rs7781964 | ALM | myopia | 0.026 | -0.022 | 0.002 | 0.043 | 1.52E-27 | 0.605 | TRUE |
| rs778384 | ALM | myopia | 0.028 | -0.007 | 0.002 | 0.040 | 7.21E-38 | 0.870 | TRUE |
| rs7816345 | ALM | myopia | 0.026 | -0.048 | 0.003 | 0.047 | 1.98E-24 | 0.310 | TRUE |
| rs78180894 | ALM | myopia | 0.034 | -0.120 | 0.004 | 0.123 | 6.53E-20 | 0.329 | TRUE |
| rs78378222 | ALM | myopia | -0.138 | 0.107 | 0.009 | 0.125 | 1.16E-56 | 0.393 | TRUE |
| rs78444298 | ALM | myopia | -0.047 | 0.049 | 0.007 | 0.126 | 8.02E-12 | 0.698 | TRUE |
| rs78457529 | ALM | myopia | -0.090 | 0.082 | 0.009 | 0.123 | 9.35E-25 | 0.503 | TRUE |
| rs7858712 | ALM | myopia | -0.035 | 0.052 | 0.003 | 0.060 | 1.87E-24 | 0.383 | TRUE |
| rs7910211 | ALM | myopia | -0.018 | 0.049 | 0.003 | 0.040 | 1.69E-11 | 0.216 | TRUE |
| rs7952436 | ALM | myopia | -0.045 | -0.094 | 0.003 | 0.073 | 1.69E-40 | 0.201 | TRUE |
| rs79680939 | ALM | myopia | 0.029 | -0.071 | 0.005 | 0.107 | 2.89E-10 | 0.507 | TRUE |
| rs7968719 | ALM | myopia | 0.013 | -0.046 | 0.002 | 0.033 | 1.76E-12 | 0.168 | FALSE |
| rs7971536 | ALM | myopia | -0.019 | -0.043 | 0.002 | 0.033 | 1.78E-24 | 0.196 | FALSE |
| rs798528 | ALM | myopia | 0.036 | -0.014 | 0.002 | 0.034 | 2.89E-71 | 0.680 | TRUE |
| rs80142996 | ALM | myopia | -0.026 | -0.047 | 0.004 | 0.089 | 5.51E-12 | 0.592 | TRUE |
| rs8017006 | ALM | myopia | -0.012 | -0.070 | 0.002 | 0.037 | 1.06E-09 | 0.057 | TRUE |
| rs8018486 | ALM | myopia | 0.014 | 0.023 | 0.002 | 0.039 | 8.92E-09 | 0.553 | TRUE |
| rs8019890 | ALM | myopia | 0.025 | 0.052 | 0.002 | 0.033 | 1.53E-39 | 0.113 | TRUE |
| rs80280630 | ALM | myopia | -0.017 | -0.107 | 0.003 | 0.061 | 2.14E-08 | 0.078 | TRUE |
| rs8042545 | ALM | myopia | 0.029 | 0.060 | 0.002 | 0.039 | 6.75E-39 | 0.126 | TRUE |
| rs8054549 | ALM | myopia | -0.025 | 0.030 | 0.002 | 0.033 | 7.63E-40 | 0.367 | TRUE |
| rs8064946 | ALM | myopia | -0.020 | -0.071 | 0.003 | 0.065 | 8.03E-11 | 0.275 | TRUE |
| rs8077636 | ALM | myopia | -0.019 | 0.074 | 0.002 | 0.034 | 5.24E-24 | 0.029 | TRUE |
| rs8084413 | ALM | myopia | -0.013 | -0.003 | 0.002 | 0.033 | 2.32E-11 | 0.934 | TRUE |
| rs8099461 | ALM | myopia | 0.025 | 0.221 | 0.004 | 0.080 | 1.97E-09 | 0.006 | TRUE |
| rs8107967 | ALM | myopia | 0.017 | 0.029 | 0.002 | 0.033 | 5.29E-20 | 0.366 | TRUE |
| rs8176632 | ALM | myopia | -0.016 | -0.011 | 0.003 | 0.039 | 1.67E-08 | 0.773 | TRUE |
| rs8180765 | ALM | myopia | 0.015 | 0.012 | 0.002 | 0.037 | 5.20E-11 | 0.737 | TRUE |
| rs853168 | ALM | myopia | 0.016 | 0.026 | 0.002 | 0.041 | 1.33E-12 | 0.526 | TRUE |
| rs876122 | ALM | myopia | -0.016 | -0.019 | 0.003 | 0.045 | 2.32E-08 | 0.676 | TRUE |
| rs8904 | ALM | myopia | -0.016 | 0.030 | 0.002 | 0.033 | 4.16E-15 | 0.365 | TRUE |
| rs894736 | ALM | myopia | 0.017 | -0.101 | 0.002 | 0.033 | 3.32E-18 | 0.002 | TRUE |
| rs905938 | ALM | myopia | -0.039 | 0.065 | 0.002 | 0.039 | 1.55E-78 | 0.095 | TRUE |
| rs921142 | ALM | myopia | 0.011 | -0.038 | 0.002 | 0.033 | 5.15E-09 | 0.248 | TRUE |
| rs9288695 | ALM | myopia | 0.013 | -0.002 | 0.002 | 0.039 | 3.36E-08 | 0.950 | TRUE |
| rs9344126 | ALM | myopia | 0.019 | 0.048 | 0.002 | 0.033 | 2.10E-22 | 0.150 | TRUE |
| rs9353118 | ALM | myopia | -0.011 | -0.083 | 0.002 | 0.037 | 3.80E-08 | 0.027 | TRUE |
| rs9376478 | ALM | myopia | -0.019 | -0.020 | 0.002 | 0.045 | 2.80E-17 | 0.654 | TRUE |
| rs9385002 | ALM | myopia | 0.015 | -0.038 | 0.002 | 0.036 | 2.36E-11 | 0.285 | TRUE |
| rs9388490 | ALM | myopia | 0.046 | -0.048 | 0.002 | 0.033 | 1.33E-130 | 0.142 | TRUE |
| rs9391254 | ALM | myopia | 0.017 | 0.080 | 0.002 | 0.035 | 1.04E-16 | 0.023 | TRUE |
| rs945508 | ALM | myopia | 0.013 | 0.021 | 0.002 | 0.033 | 1.62E-11 | 0.515 | TRUE |
| rs947800 | ALM | myopia | -0.033 | 0.067 | 0.005 | 0.076 | 8.57E-11 | 0.380 | TRUE |
| rs9479012 | ALM | myopia | -0.026 | 0.016 | 0.003 | 0.056 | 2.06E-16 | 0.778 | TRUE |
| rs9492799 | ALM | myopia | 0.018 | -0.015 | 0.003 | 0.046 | 2.56E-12 | 0.749 | TRUE |
| rs9513510 | ALM | myopia | -0.018 | -0.027 | 0.002 | 0.035 | 6.75E-18 | 0.430 | TRUE |
| rs951366 | ALM | myopia | -0.021 | -0.022 | 0.002 | 0.034 | 3.86E-27 | 0.523 | TRUE |
| rs9525326 | ALM | myopia | 0.018 | 0.036 | 0.002 | 0.042 | 1.77E-14 | 0.382 | TRUE |
| rs9579402 | ALM | myopia | -0.020 | 0.028 | 0.004 | 0.050 | 6.63E-09 | 0.580 | TRUE |
| rs9590328 | ALM | myopia | -0.015 | 0.004 | 0.003 | 0.048 | 1.46E-08 | 0.929 | TRUE |
| rs9610447 | ALM | myopia | 0.015 | -0.023 | 0.002 | 0.039 | 4.88E-12 | 0.560 | TRUE |
| rs963317 | ALM | myopia | 0.014 | 0.011 | 0.002 | 0.035 | 1.05E-11 | 0.755 | TRUE |
| rs9634212 | ALM | myopia | 0.047 | -0.031 | 0.002 | 0.038 | 3.36E-93 | 0.407 | TRUE |
| rs9636364 | ALM | myopia | 0.011 | 0.017 | 0.002 | 0.033 | 7.06E-09 | 0.612 | TRUE |
| rs9647379 | ALM | myopia | 0.022 | -0.024 | 0.002 | 0.033 | 1.10E-29 | 0.460 | FALSE |
| rs9659061 | ALM | myopia | -0.020 | 0.081 | 0.002 | 0.033 | 1.99E-25 | 0.015 | TRUE |
| rs9696116 | ALM | myopia | 0.019 | 0.040 | 0.002 | 0.035 | 5.46E-21 | 0.257 | TRUE |
| rs9784904 | ALM | myopia | 0.022 | -0.064 | 0.003 | 0.038 | 7.05E-17 | 0.097 | TRUE |
| rs9807032 | ALM | myopia | 0.024 | 0.080 | 0.002 | 0.044 | 1.52E-23 | 0.068 | TRUE |
| rs9809116 | ALM | myopia | 0.016 | 0.034 | 0.002 | 0.035 | 3.73E-17 | 0.332 | TRUE |
| rs9817452 | ALM | myopia | 0.017 | -0.041 | 0.002 | 0.035 | 3.81E-18 | 0.241 | TRUE |
| rs9828525 | ALM | myopia | 0.012 | -0.006 | 0.002 | 0.033 | 1.91E-10 | 0.855 | TRUE |
| rs9832919 | ALM | myopia | 0.018 | -0.009 | 0.002 | 0.034 | 3.55E-19 | 0.802 | TRUE |
| rs9838614 | ALM | myopia | 0.019 | 0.066 | 0.002 | 0.033 | 2.10E-22 | 0.047 | TRUE |
| rs985136 | ALM | myopia | -0.014 | 0.019 | 0.002 | 0.035 | 5.20E-12 | 0.595 | FALSE |
| rs9853018 | ALM | myopia | 0.047 | 0.104 | 0.002 | 0.033 | 1.58E-134 | 0.001 | TRUE |
| rs9861931 | ALM | myopia | 0.011 | 0.052 | 0.002 | 0.033 | 7.06E-09 | 0.116 | FALSE |
| rs9894577 | ALM | myopia | -0.031 | 0.017 | 0.002 | 0.035 | 3.47E-54 | 0.627 | TRUE |
| rs9905385 | ALM | myopia | 0.034 | 0.058 | 0.002 | 0.037 | 1.92E-64 | 0.113 | TRUE |
| rs9910161 | ALM | myopia | -0.016 | -0.091 | 0.002 | 0.037 | 1.22E-14 | 0.015 | TRUE |
| rs9931073 | ALM | myopia | -0.015 | 0.030 | 0.002 | 0.033 | 1.91E-15 | 0.357 | FALSE |
| rs994533 | ALM | myopia | -0.031 | -0.021 | 0.002 | 0.033 | 3.32E-55 | 0.517 | FALSE |
| rs9957318 | ALM | myopia | -0.019 | -0.015 | 0.002 | 0.035 | 8.76E-21 | 0.672 | TRUE |
| rs10801580 | FA BMD | myopia | 0.089 | -0.031 | 0.019 | 0.036 | 2.58E-06 | 0.387 | TRUE |
| rs115419274 | FA BMD | myopia | 0.451 | 0.003 | 0.097 | 0.098 | 4.90E-06 | 0.978 | TRUE |
| rs1336819 | FA BMD | myopia | -0.234 | -0.031 | 0.049 | 0.072 | 3.17E-06 | 0.672 | TRUE |
| rs13423976 | FA BMD | myopia | -0.098 | -0.054 | 0.017 | 0.036 | 2.30E-08 | 0.136 | TRUE |
| rs145750983 | FA BMD | myopia | -0.299 | 0.000 | 0.063 | 0.186 | 2.87E-06 | 0.999 | TRUE |
| rs1982763 | FA BMD | myopia | 0.079 | 0.043 | 0.016 | 0.033 | 1.11E-06 | 0.192 | TRUE |
| rs35936330 | FA BMD | myopia | 0.095 | -0.041 | 0.020 | 0.040 | 3.30E-06 | 0.310 | TRUE |
| rs489247 | FA BMD | myopia | -0.086 | 0.038 | 0.018 | 0.037 | 2.90E-06 | 0.314 | TRUE |
| rs58715565 | FA BMD | myopia | -0.174 | 0.115 | 0.035 | 0.080 | 1.24E-06 | 0.151 | TRUE |
| rs61066067 | FA BMD | myopia | -0.138 | 0.038 | 0.027 | 0.057 | 5.87E-07 | 0.502 | TRUE |
| rs61904896 | FA BMD | myopia | 0.154 | 0.052 | 0.031 | 0.055 | 8.45E-07 | 0.349 | TRUE |
| rs6894139 | FA BMD | myopia | -0.089 | -0.018 | 0.016 | 0.033 | 2.52E-08 | 0.594 | TRUE |
| rs7216991 | FA BMD | myopia | -0.074 | -0.050 | 0.016 | 0.033 | 4.87E-06 | 0.128 | TRUE |
| rs7555157 | FA BMD | myopia | 0.084 | 0.025 | 0.018 | 0.039 | 3.75E-06 | 0.525 | TRUE |
| rs7776725 | FA BMD | myopia | 0.186 | -0.062 | 0.017 | 0.037 | 1.21E-25 | 0.094 | TRUE |
| rs7815608 | FA BMD | myopia | -0.081 | 0.056 | 0.017 | 0.038 | 4.34E-06 | 0.140 | TRUE |
| rs10170839 | FN BMD | myopia | -0.059 | 0.033 | 0.008 | 0.033 | 1.20E-14 | 0.306 | TRUE |
| rs10794639 | FN BMD | myopia | -0.051 | 0.035 | 0.008 | 0.033 | 3.30E-11 | 0.290 | TRUE |
| rs10946458 | FN BMD | myopia | -0.045 | 0.039 | 0.008 | 0.035 | 3.63E-08 | 0.258 | TRUE |
| rs11652763 | FN BMD | myopia | 0.084 | -0.005 | 0.013 | 0.046 | 1.09E-10 | 0.919 | TRUE |
| rs1366594 | FN BMD | myopia | -0.079 | -0.020 | 0.008 | 0.033 | 5.44E-25 | 0.553 | TRUE |
| rs1485307 | FN BMD | myopia | 0.062 | 0.033 | 0.008 | 0.033 | 2.49E-15 | 0.312 | TRUE |
| rs1785493 | FN BMD | myopia | -0.045 | -0.005 | 0.008 | 0.041 | 4.06E-08 | 0.895 | TRUE |
| rs2566752 | FN BMD | myopia | 0.062 | -0.009 | 0.008 | 0.034 | 3.65E-15 | 0.788 | TRUE |
| rs2741856 | FN BMD | myopia | 0.088 | -0.009 | 0.014 | 0.050 | 1.34E-09 | 0.859 | TRUE |
| rs3779381 | FN BMD | myopia | 0.058 | -0.044 | 0.009 | 0.038 | 2.87E-11 | 0.243 | TRUE |
| rs436448 | FN BMD | myopia | -0.064 | 0.039 | 0.008 | 0.033 | 1.56E-16 | 0.238 | TRUE |
| rs4448201 | FN BMD | myopia | -0.066 | 0.024 | 0.008 | 0.037 | 4.37E-16 | 0.509 | TRUE |
| rs4759320 | FN BMD | myopia | -0.045 | -0.081 | 0.008 | 0.034 | 3.33E-08 | 0.017 | TRUE |
| rs7108738 | FN BMD | myopia | 0.083 | 0.039 | 0.010 | 0.036 | 8.07E-17 | 0.273 | TRUE |
| rs71390846 | FN BMD | myopia | -0.059 | -0.024 | 0.010 | 0.040 | 3.16E-09 | 0.551 | TRUE |
| rs7209460 | FN BMD | myopia | -0.051 | -0.042 | 0.008 | 0.035 | 1.35E-09 | 0.235 | TRUE |
| rs7524102 | FN BMD | myopia | 0.084 | -0.040 | 0.010 | 0.057 | 7.36E-17 | 0.487 | TRUE |
| rs9478217 | FN BMD | myopia | -0.053 | 0.045 | 0.008 | 0.033 | 1.23E-11 | 0.181 | TRUE |
| rs1023940 | LS BMD | myopia | 0.065 | 0.007 | 0.009 | 0.033 | 6.47E-13 | 0.843 | TRUE |
| rs11002249 | LS BMD | myopia | 0.070 | 0.066 | 0.011 | 0.047 | 1.01E-09 | 0.161 | TRUE |
| rs11024028 | LS BMD | myopia | 0.066 | -0.084 | 0.011 | 0.047 | 1.44E-08 | 0.071 | TRUE |
| rs11680288 | LS BMD | myopia | 0.054 | -0.021 | 0.009 | 0.033 | 3.12E-09 | 0.517 | TRUE |
| rs11692564 | LS BMD | myopia | 0.238 | 0.093 | 0.039 | 0.112 | 4.10E-09 | 0.407 | TRUE |
| rs13046645 | LS BMD | myopia | -0.056 | 0.014 | 0.010 | 0.042 | 2.92E-08 | 0.748 | TRUE |
| rs1357651 | LS BMD | myopia | -0.068 | -0.013 | 0.009 | 0.037 | 3.75E-13 | 0.726 | TRUE |
| rs2220189 | LS BMD | myopia | 0.083 | -0.035 | 0.009 | 0.033 | 4.25E-20 | 0.279 | FALSE |
| rs2235811 | LS BMD | myopia | -0.054 | -0.024 | 0.009 | 0.033 | 4.66E-09 | 0.458 | TRUE |
| rs2291467 | LS BMD | myopia | -0.077 | -0.026 | 0.010 | 0.045 | 9.64E-14 | 0.566 | TRUE |
| rs2566752 | LS BMD | myopia | 0.083 | -0.009 | 0.009 | 0.034 | 1.49E-19 | 0.788 | TRUE |
| rs35681117 | LS BMD | myopia | 0.055 | 0.041 | 0.010 | 0.036 | 2.39E-08 | 0.250 | TRUE |
| rs401680 | LS BMD | myopia | -0.057 | -0.045 | 0.009 | 0.033 | 3.70E-10 | 0.179 | FALSE |
| rs6965122 | LS BMD | myopia | -0.062 | -0.018 | 0.009 | 0.036 | 7.40E-11 | 0.622 | TRUE |
| rs71390846 | LS BMD | myopia | -0.064 | -0.024 | 0.011 | 0.040 | 3.80E-08 | 0.551 | TRUE |
| rs73326583 | LS BMD | myopia | 0.072 | 0.006 | 0.012 | 0.049 | 2.83E-09 | 0.911 | TRUE |
| rs7524102 | LS BMD | myopia | 0.090 | -0.040 | 0.011 | 0.057 | 2.41E-14 | 0.487 | TRUE |
| rs7807953 | LS BMD | myopia | 0.075 | -0.054 | 0.010 | 0.038 | 4.11E-14 | 0.158 | TRUE |
| rs78667121 | LS BMD | myopia | 0.150 | 0.055 | 0.026 | 0.078 | 1.21E-08 | 0.481 | TRUE |
| rs884205 | LS BMD | myopia | -0.062 | 0.054 | 0.010 | 0.037 | 2.77E-09 | 0.136 | TRUE |
| rs894738 | LS BMD | myopia | -0.063 | 0.097 | 0.009 | 0.033 | 2.00E-11 | 0.004 | TRUE |
| rs9533094 | LS BMD | myopia | -0.083 | -0.029 | 0.009 | 0.033 | 2.80E-20 | 0.384 | TRUE |
| rs9921222 | LS BMD | myopia | -0.053 | 0.036 | 0.009 | 0.033 | 3.16E-09 | 0.266 | TRUE |

BMD: bone mineral density; ALM: appendicular lean mass; FA: forearm; FN: femoral neck; LS: lumbar spine; IVs: instrumental variables
