# Supplementary material for: Osteoporosis and sarcopenia-related traits: A bi-directional Mendelian randomization study
Source: Front Endocrinol (Lausanne). 2022 Sep 14;13:975647. doi: 10.3389/fendo.2022.975647 (PMC9515352; doi:10.3389/fendo.2022.975647)
Supplement: Supplementary file 7 [file Table_7.docx]

Supplementary Table 7. Two-sample mendelian randomization analysis of negative control (myopia)

| Exposure | outcome | method | n. Ivs | P.value | beta (95% CI) |
| --- | --- | --- | --- | --- | --- |
| low‐grip strength | myopia | Inverse variance weighted | 13 | 0.148 | -0.285(-0.671,0.100) |
| low‐grip strength | myopia | Weighted median | 13 | 0.141 | -0.411(-0.959,0.136) |
| low‐grip strength | myopia | Robust adjusted profile score (RAPS) | 13 | 0.343 | -0.238(-0.729,0.253) |
| ALM | myopia | Inverse variance weighted | 574 | 0.542 | -0.049(-0.207,0.109) |
| ALM | myopia | Weighted median | 574 | 0.057 | -0.232(-0.472,0.007) |
| ALM | myopia | Robust adjusted profile score (RAPS) | 574 | 0.303 | -0.084(-0.242,0.075) |
| FA BMD | myopia | Inverse variance weighted | 16 | 0.625 | -0.043(-0.215,0.129) |
| FA BMD | myopia | Weighted median | 16 | 0.977 | -0.004(-0.250,0.257) |
| FA BMD | myopia | Robust adjusted profile score (RAPS) | 16 | 0.628 | -0.046(-0.233,0.140) |
| FN BMD | myopia | Inverse variance weighted | 18 | 0.726 | -0.048(-0.317,0.221) |
| FN BMD | myopia | Weighted median | 18 | 0.622 | -0.097(-0.483,0.289) |
| FN BMD | myopia | Robust adjusted profile score (RAPS) | 18 | 0.592 | -0.080(-0.374,0.213) |
| LS BMD | myopia | Inverse variance weighted | 21 | 0.622 | -0.060(-0.298,0.178) |
| LS BMD | myopia | Weighted median | 21 | 0.371 | 0.156(-0.326,0.206) |
| LS BMD | myopia | Robust adjusted profile score (RAPS) | 21 | 0.96 | -0.007(-0.266,0.252) |

BMD: bone mineral density; ALM: appendicular lean mass; FA: forearm; FN: femoral neck; LS: lumbar spine; IVs: instrumental variables.
